# Supplementary material for: Electrochromic windows with fast response and wide dynamic range for visible-light modulation without traditional electrodes
Source: Nat Commun. 2024 Jul 20;15:6110. doi: 10.1038/s41467-024-50542-3 (PMC11271603; doi:10.1038/s41467-024-50542-3)
Supplement: Supplementary file 1 — Supplementary Information [file 41467_2024_50542_MOESM1_ESM.pdf]

Supplementary Information for

Electrochromic windows with fast response and wide dynamic range for visible-light modulation without traditional electrodes

Zhuofei Jia[1, #], Yiming Sui[1, #], Long Qian[1, #], Xi Ren[1], Yunxiang Zhao[1], Rui Yao[1], Lumeng Wang[1], Dongliang Chao[2], Cheng Yang[1, ✉]

Affiliations:

[1]Institute of Materials Research, Tsinghua Shenzhen International Graduate School, Tsinghua University, Shenzhen 518055, P.R. China.

[2]Laboratory of Advanced Materials, Shanghai Key Laboratory of Molecular Catalysis and Innovative Materials, State Key Laboratory of Molecular Engineering of Polymers, College of Chemistry and Materials, Fudan University, Shanghai 200433, P. R. China.

[#] These authors contributed equally: Zhuofei Jia, Yiming Sui, and Long Qian.

Corresponding authors

[✉]Email: yang.cheng@sz.tsinghua.edu.cn

This PDF file includes:

Supplementary Figs. 1-35

Supplementary Tables 1-8

References

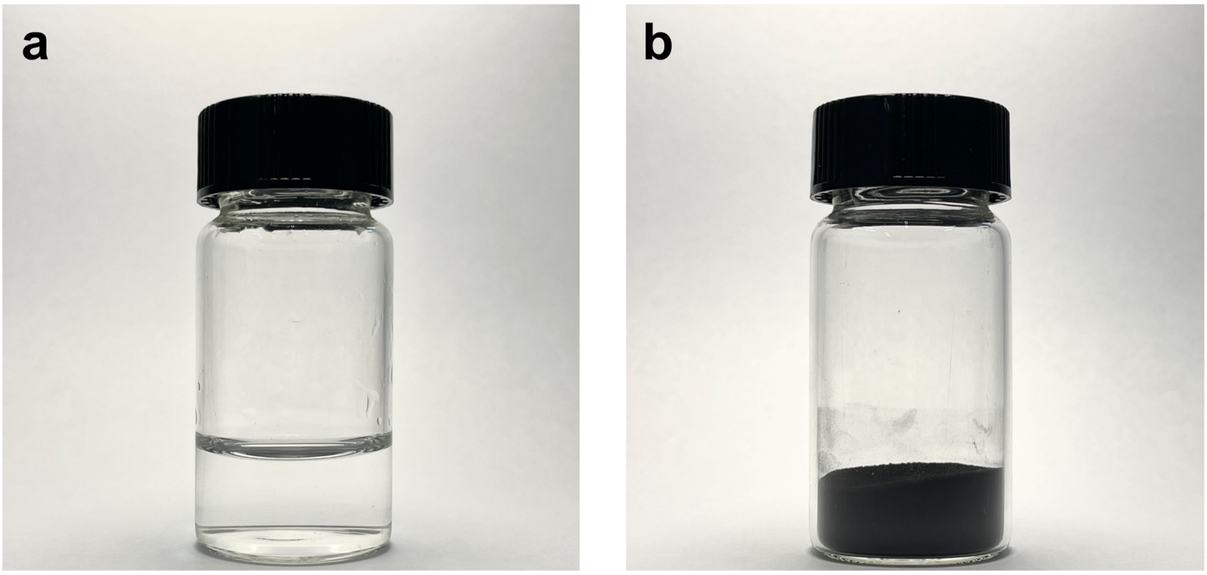

Supplementary Fig. 1. a, Photographs of 0.5 M $MnSO_4$ solution; **b,** MnO_2 powder.

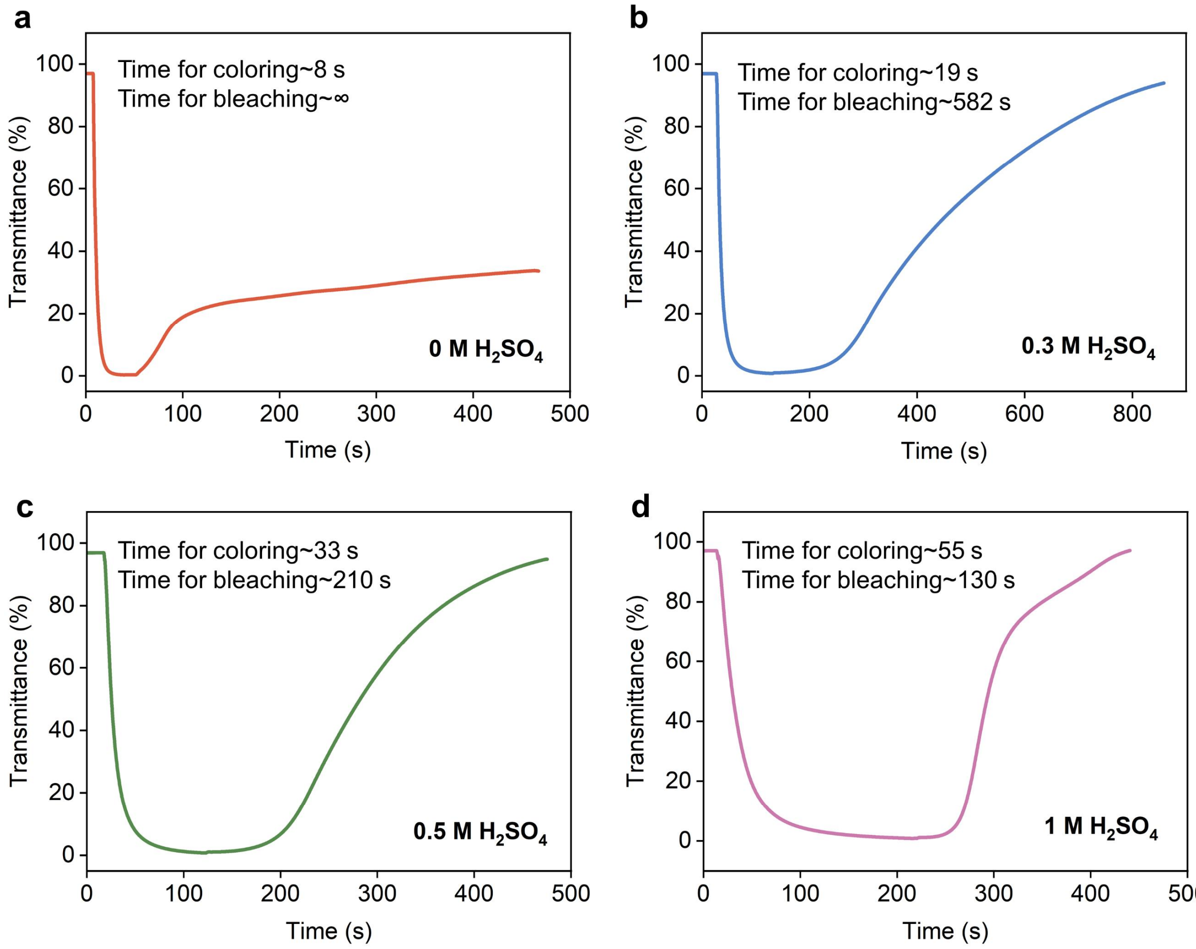

Supplementary Fig. 2. *In situ* optical transmittance profiles of the MnO_2/Mn^{2+} deposition/dissolution process in electrolytes with different acid concentrations: **a,** 0 M H_2SO_4; **b,** 0.3 M H_2SO_4; **c,** 0.5 M H_2SO_4; **d,** 1 M H_2SO_4.

To pinpoint the appropriate electrolyte, we evaluated the electrochemical and optical process of the electrolyte with different concentrations of H_2SO_4 from 0 to 1 M. Supplementary Fig. 2 presents the in situ transmittance measurements taken at a wavelength of 460 nm. Initially, a charging potential of 1.6 V was applied to achieve approximately 0% transmittance. Subsequently, a discharging potential of 0.2 V was used to facilitate the dissolution of MnO_2. When using the electrolyte without acid, MnO_2 is difficult to dissolve and the FTO electrode cannot return to the transparent state where the transmittance is about 30%. In contrast, the MnO_2/Mn^{2+} deposition/dissolution process demonstrates excellent reversibility in electrolytes with the H_2SO_4 concentrations of 0.3 M, 0.5 M, and 1 M with the transmittance through the FTO electrode, achieving about 90% at the fully bleached state. It reveals a tendency that the MnO_2 dissolution process can be accelerated by increasing the concentration of H_2SO_4 in electrolyte. For example, the bleaching time is about 582 s in electrolyte with 0.3 M H_2SO_4 (Supplementary Fig. 2b), while the bleaching time is 210 s and 130 s in electrolytes with 0.5 M and 1 M H_2SO_4 respectively (Supplementary Fig.2c, d).

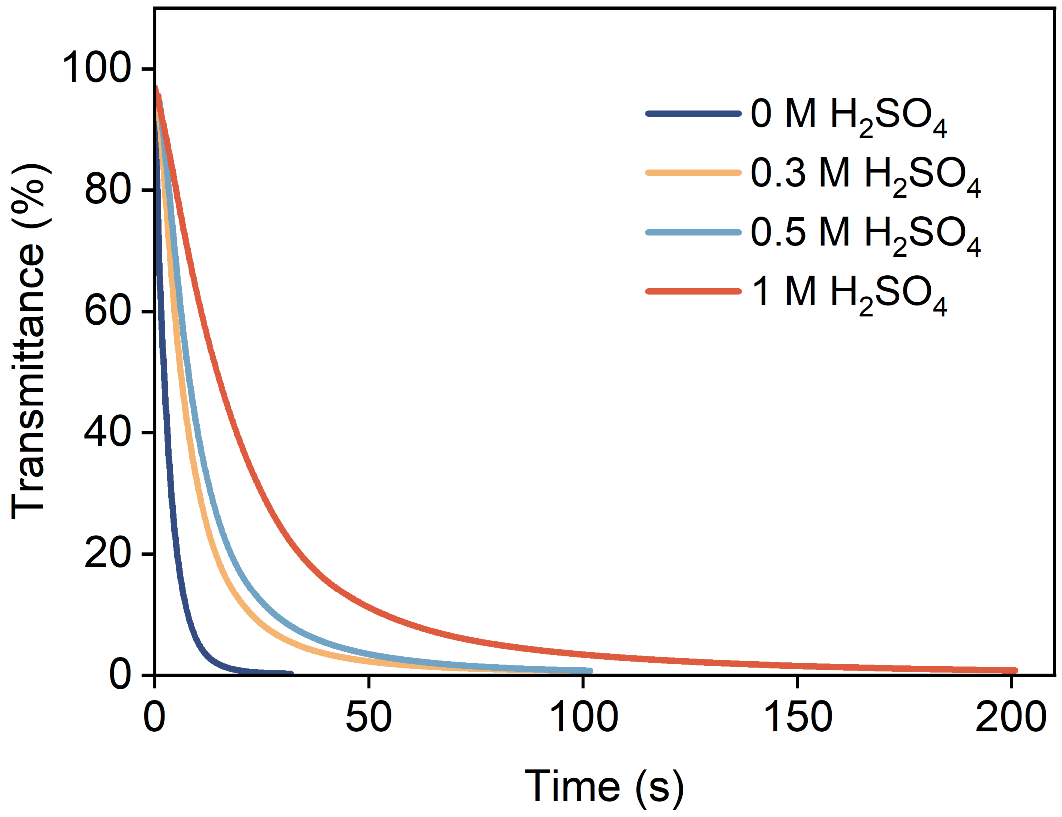

Supplementary Fig. 3. Optical transmittance at 460 nm of MnO_2 deposition as a function of time in electrolytes with different acid concentrations.

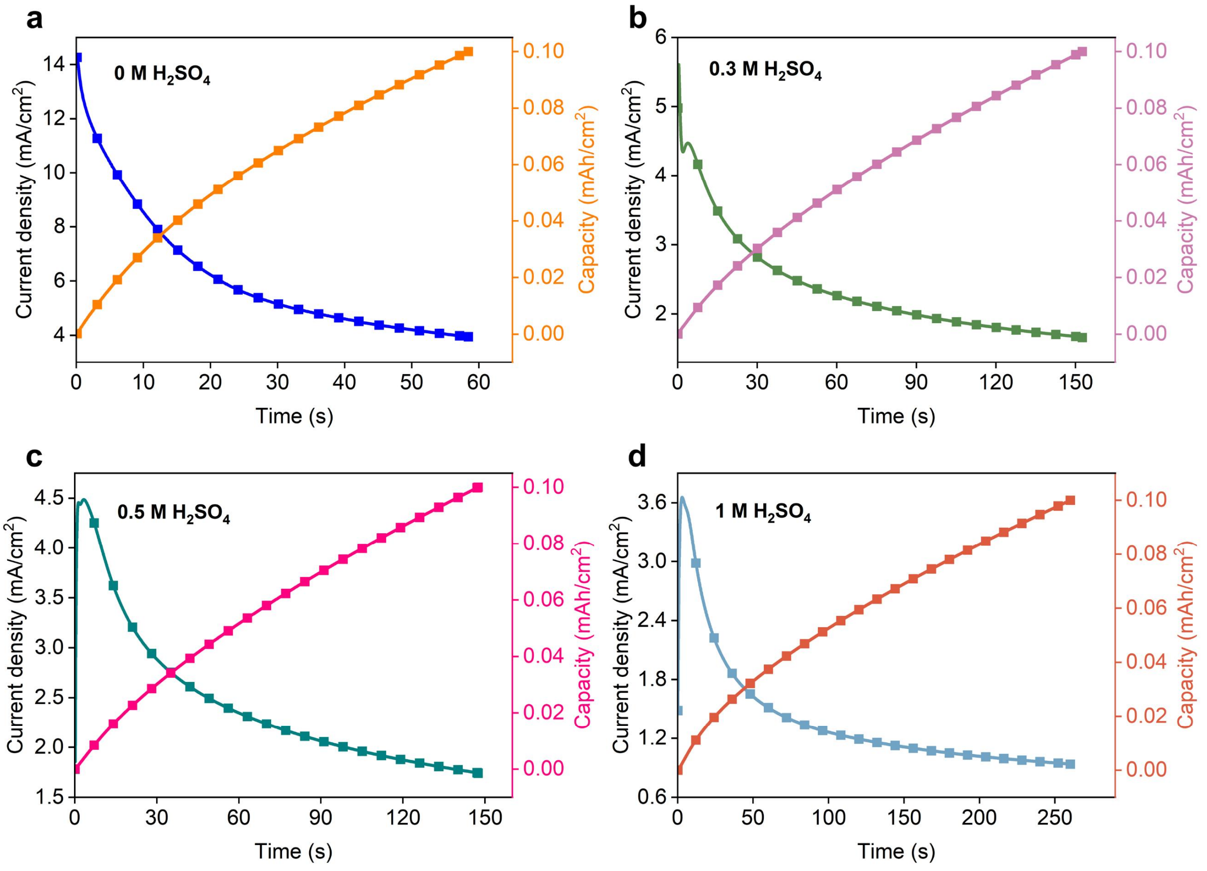

Supplementary Fig. 4. The charging current profiles of different acid concentrations in electrolytes: **a,** 0 M H_2SO_4; **b,** 0.3 M H_2SO_4; **c,** 0.5 M H_2SO_4; **d,** 1 M H_2SO_4.

Noteworthily, increasing H^+ concentration would slow down the MnO_2 deposition rate. We compared the coloring time in electrolytes with different acid concentrations and found that the addition of acid did prolong the coloring time (Supplementary Fig. 3). MnO_2 deposition current density greatly decreased when acid was added to the electrolyte and the corresponding deposition current profiles are shown in Supplementary Fig. 4. Although acidic environments would largely accelerate the MnO_2 dissolution process, it will also increase the deposition (coloring) time. Thus, considering the electrochemical and optical reversibility, as well as the response time, we optimized the electrolyte with 0.5 M H_2SO_4.

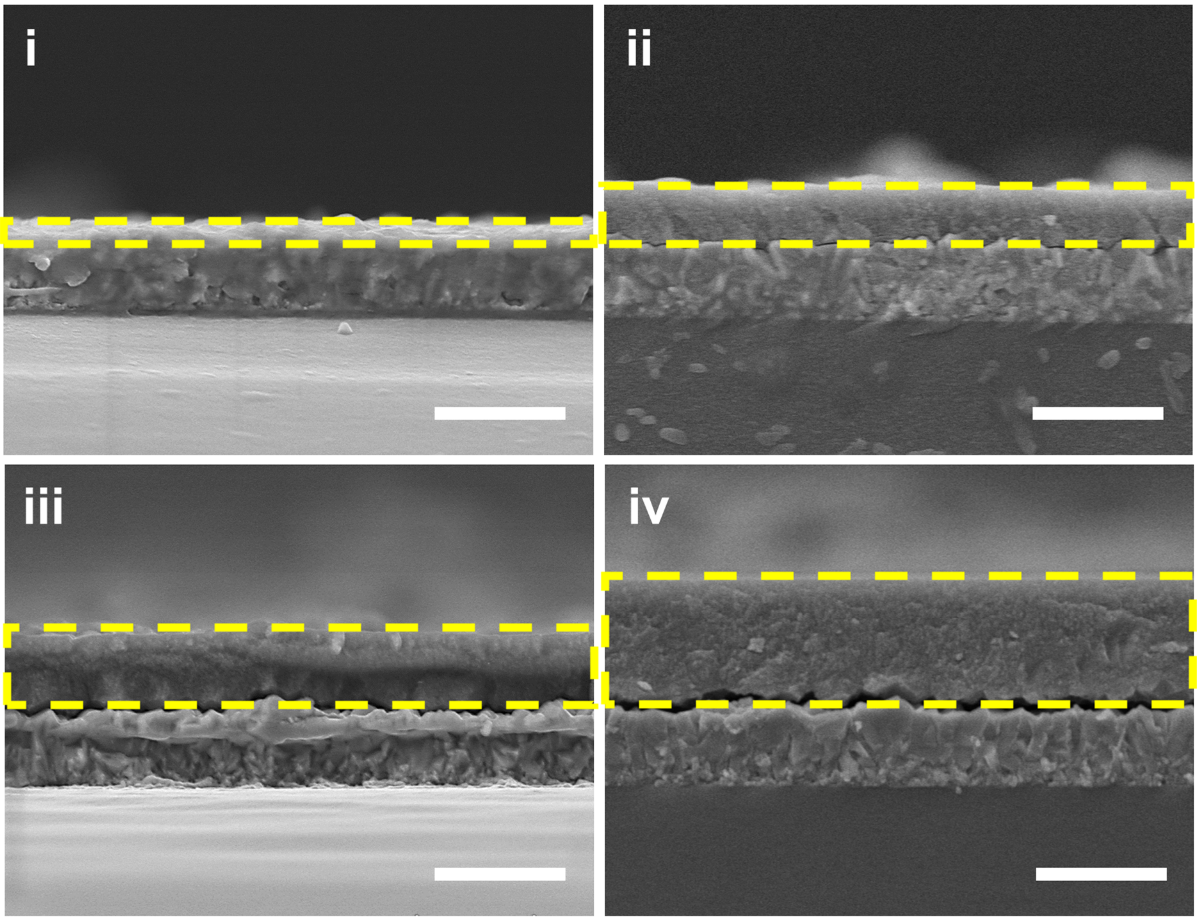

Supplementary Fig. 5. SEM images of MnO_2 deposited on the FTO cross-sectional morphology after deposition at 1.6 V vs SHE for 30 s (i), 180 s (ii), 300 s (iii), and 600 s (iv). Scale bars: 1 µm.

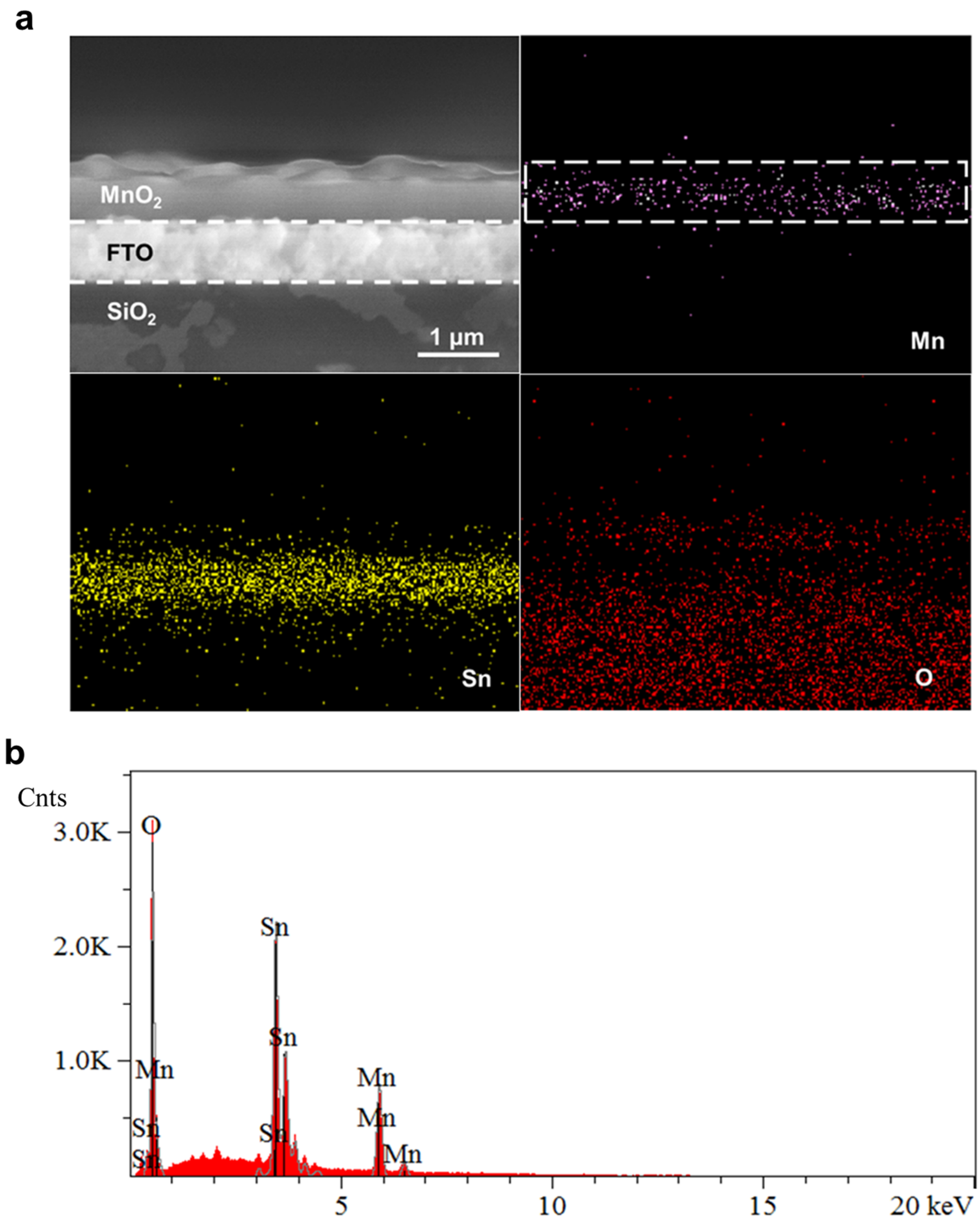

Supplementary Fig. 6. a, SEM image of cross-sectional MnO_2-deposited-on-FTO-quartz-glass and corresponding EDS mapping. **b,** EDS analysis of the same sample, verifying the existing elemental composition.

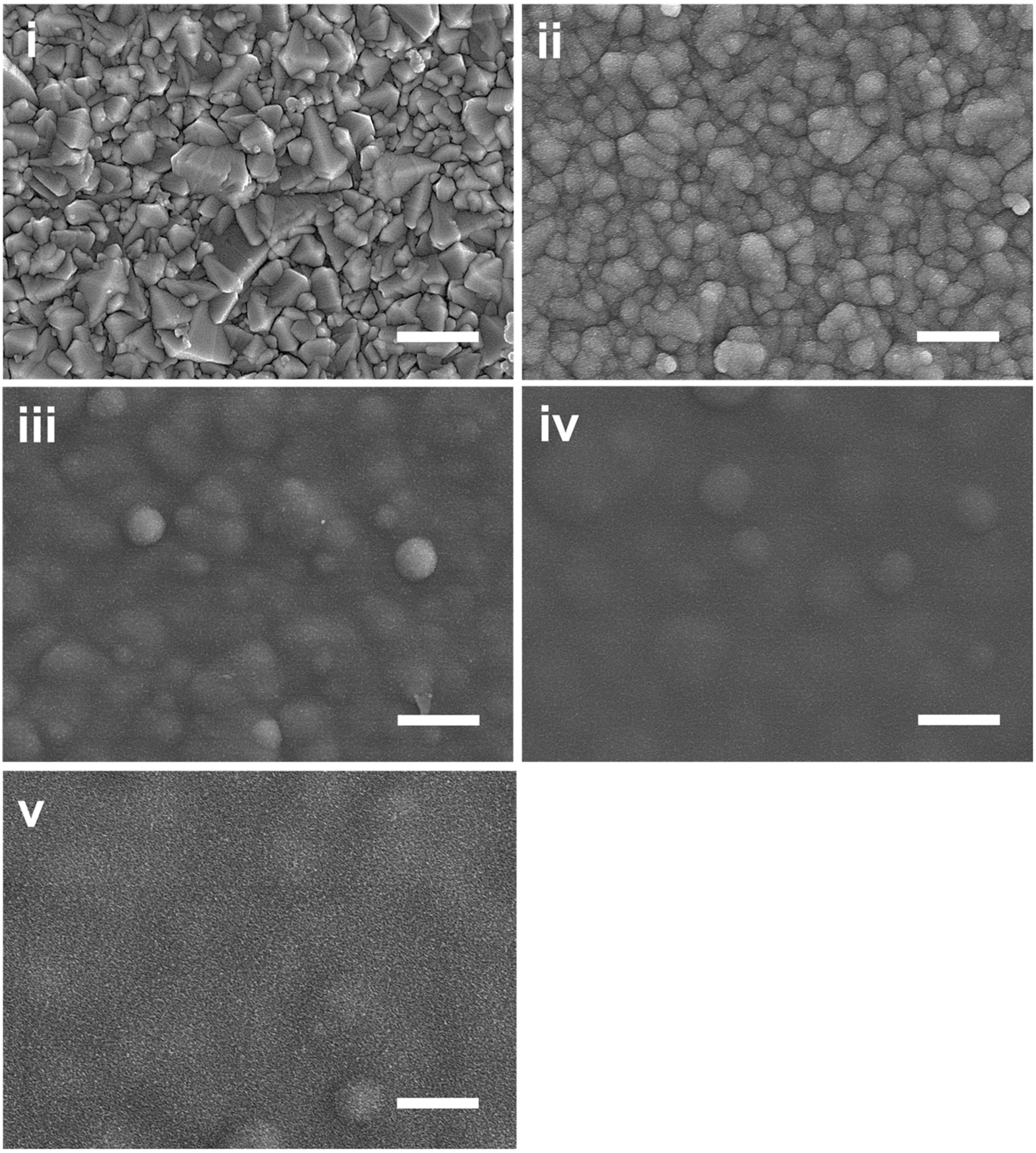

Supplementary Fig. 7. SEM images of FTO electrode (overview) before depositing MnO_2 (i) and after deposition at 1.6 V vs. SHE for 30 s (ii), 180 s (iii), 300 s (iv), and 600 s (v). Scale bars: 1 µm.

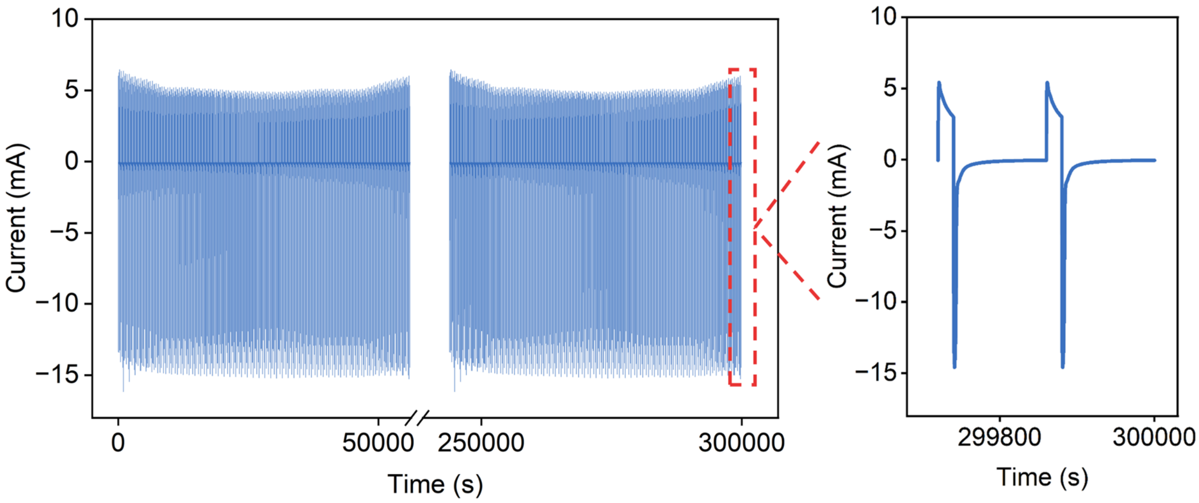

Supplementary Fig. 8. Chronoamperometry (CA) curve of MnO_2/Mn^{2+} deposition/dissolution on FTO electrode as working electrode with potential switching between 1.6 V (30 s) and 0.2 V (120 s) for 2000 times in a three-electrode configuration.

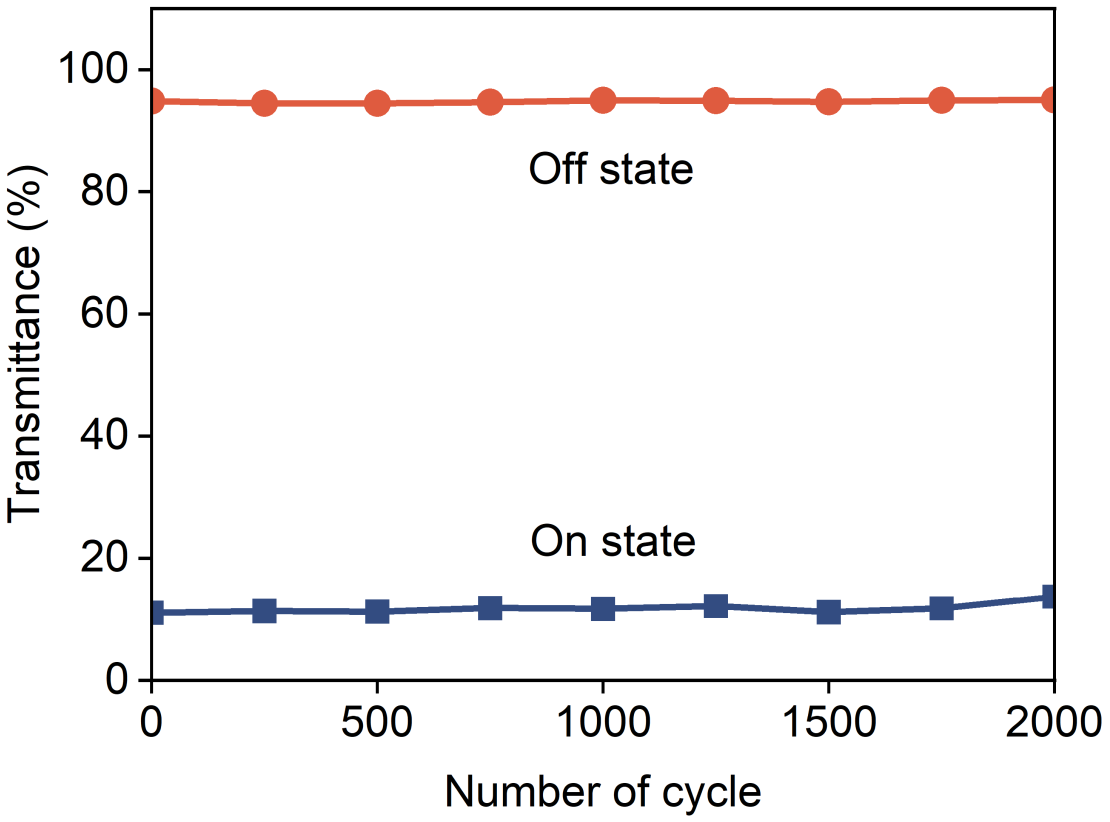

Supplementary Fig. 9. Maximum (red line) and minimum (blue line) transmission values at 460 nm during 2000 cycles of MnO_2 deposition/dissolution on FTO electrode.

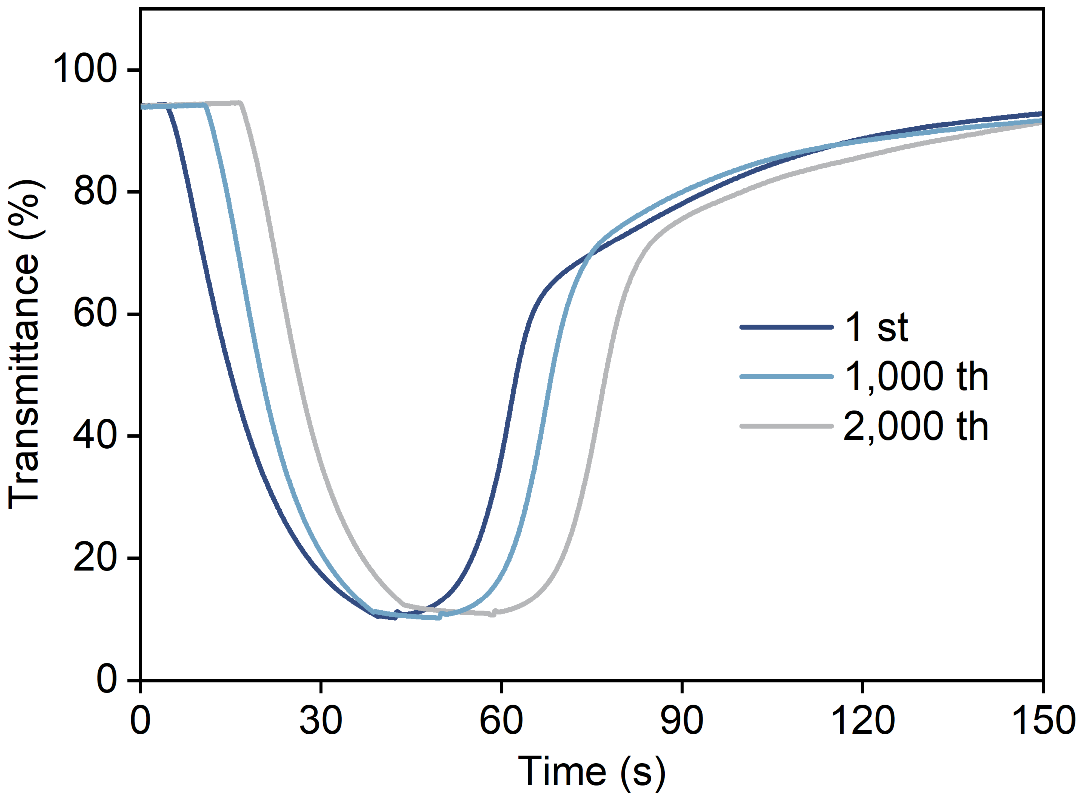

Supplementary Fig. 10. The transmittance spectra of the 1st, 1,000th, and 2,000th cycle of MnO_2/Mn^{2+} deposition/dissolution on FTO electrode.

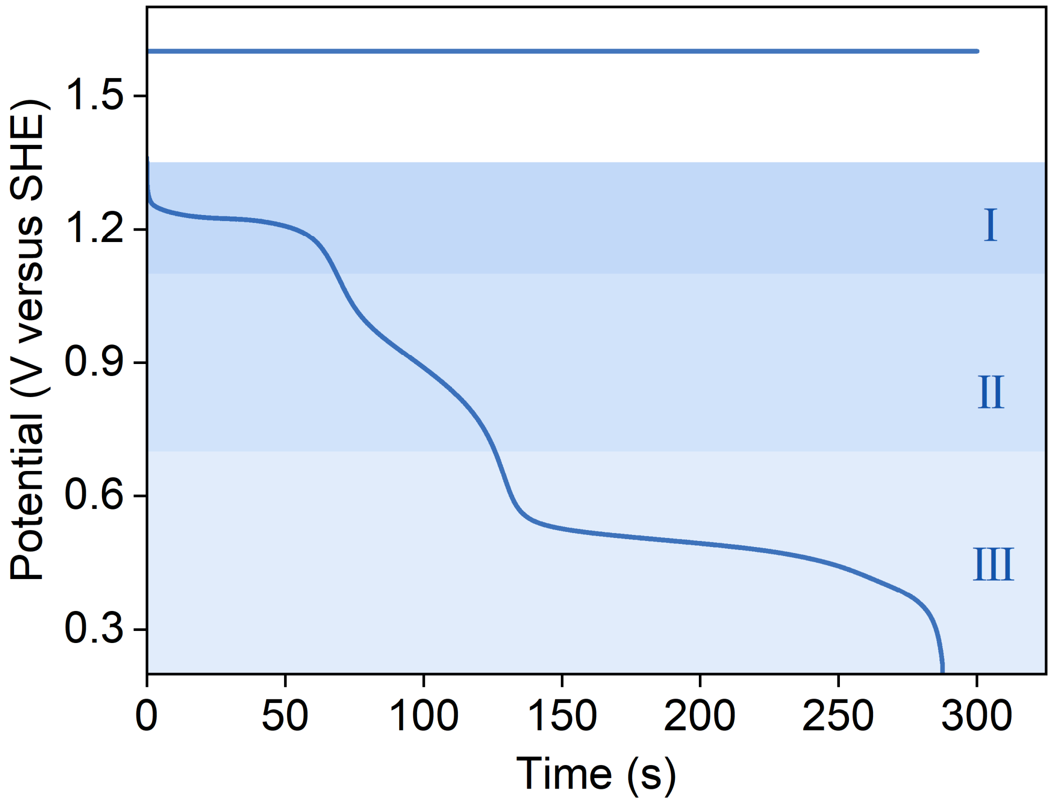

Supplementary Fig. 11. Voltage profiles of MnO_2/Mn^{2+} deposition/dissolution, where deposition at 1.6 V vs. SHE for 300 s was followed by dissolution in a galvanostatic discharge of 5 mA cm^{-2}. The three shaded areas indicate three dissolution steps corresponding with Ⅰ, Ⅱ, and Ⅲ.

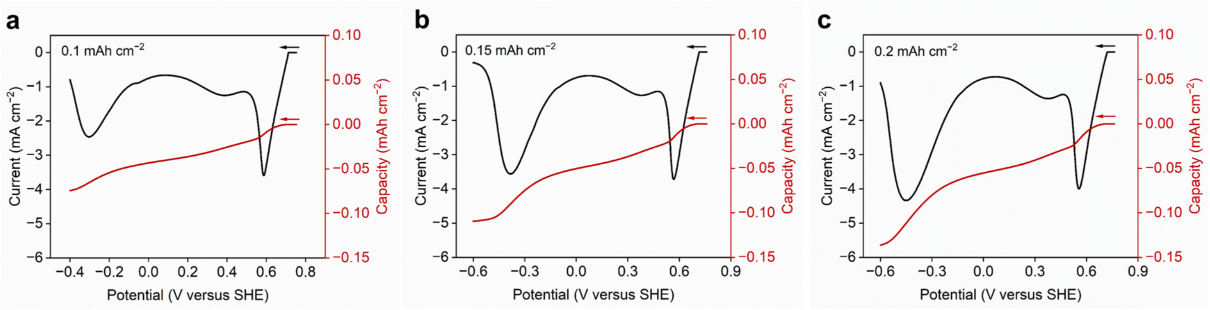

Supplementary Fig. 12. The reduction (dissolution) of MnO_2 electrodes with different mass loadings. LSV curves (black) and the corresponding discharge capacity-potential profiles (red) of the MnO_2 electrodes.

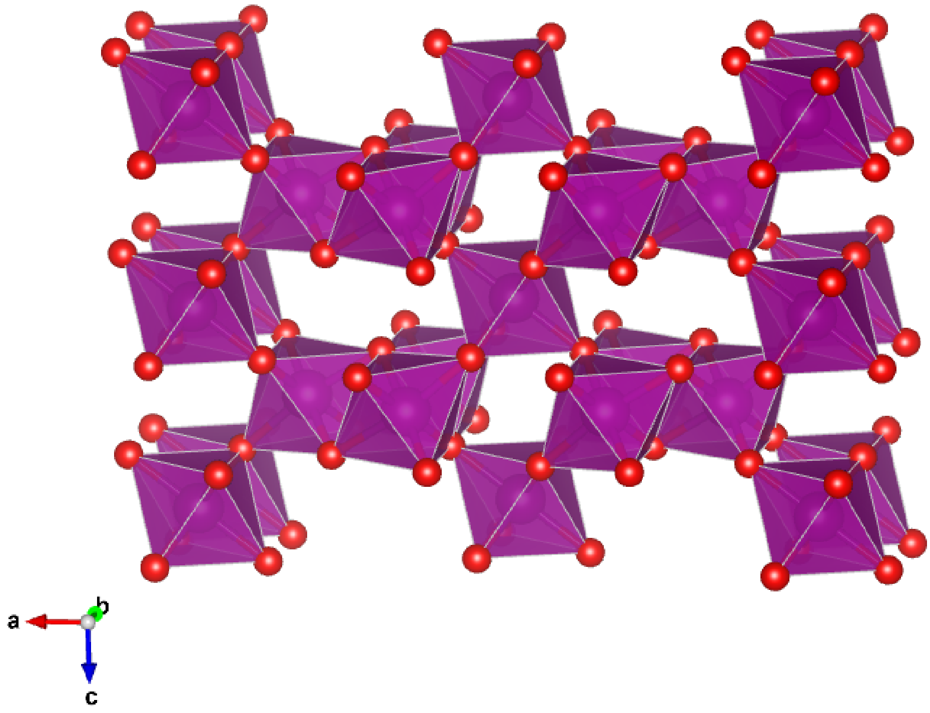

Supplementary Fig. 13. Schematic illustrating of the crystal structure of γ-MnO_2.

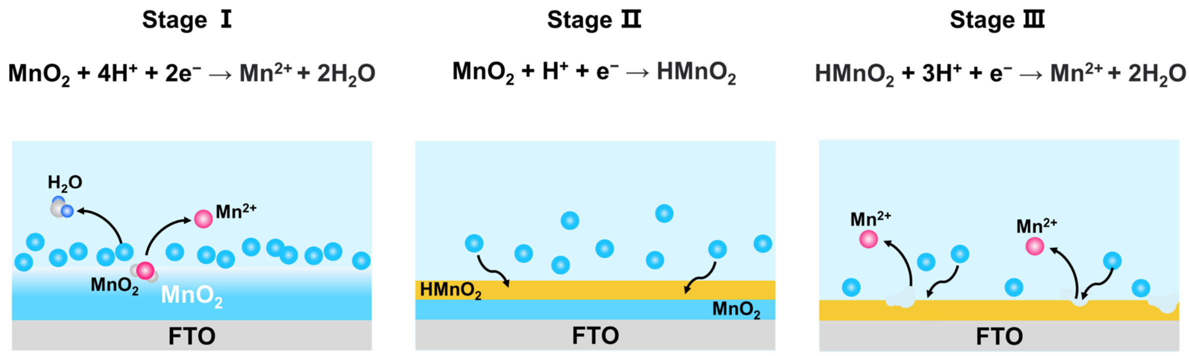

Supplementary Fig. 14. Schematic illustrations of the dissolution stages of MnO_2 on FTO electrodes. They include the dissolution of two-electron transfer at the high-voltage region and the disproportion-dissolution caused by H^+ insertion in the low-voltage region.

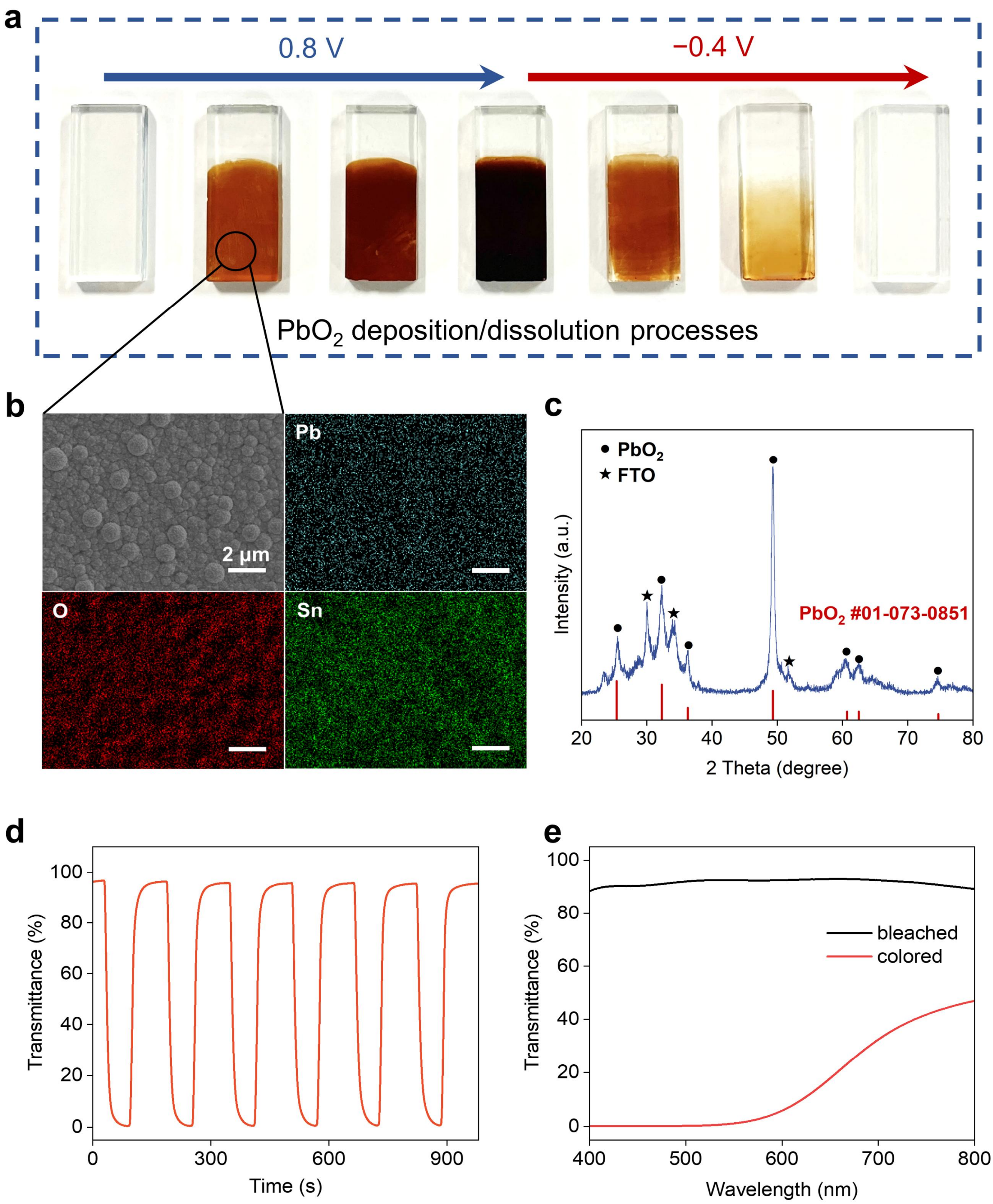

Supplementary Fig. 15. a, Photographic images of the PbO_2/FTO electrode, which show the color changing steps of the electrode sample at different deposition/dissolution stages. **b,** SEM image of PbO_2 deposited on FTO electrode and corresponding EDS mapping images. **c,** XRD pattern of the deposited PbO_2 on FTO electrode. **d,** Plot of transmittance at 460 nm as a function of time with applied deposition and dissolution potentials between 0.8 V and −0.4 V. **e,** UV-Vis transmittance spectra of the PbO_2/FTO electrode at the colored and bleached states across the visible spectrum of 400-800 nm.

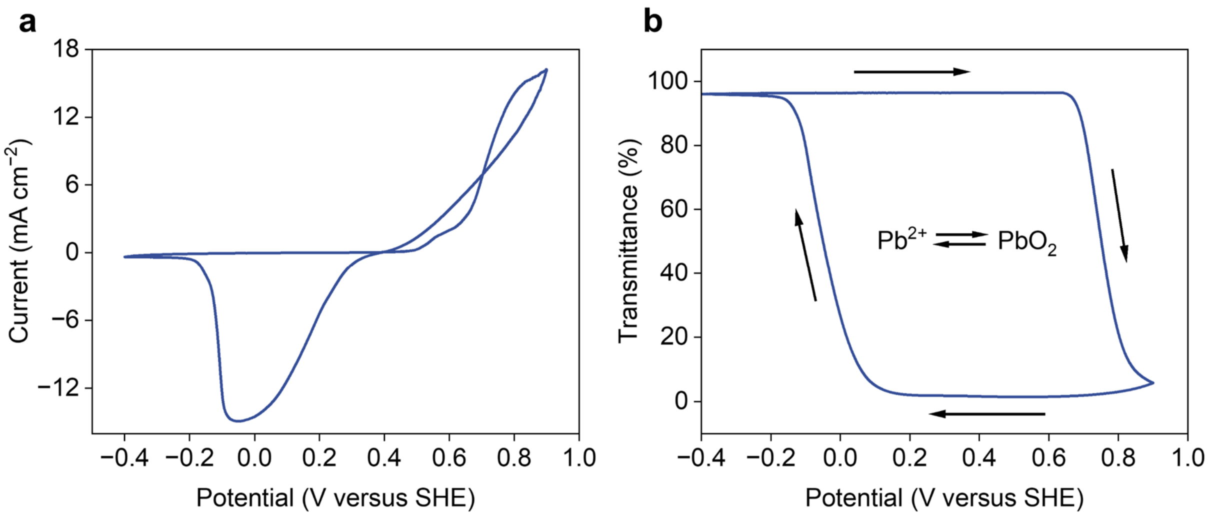

Supplementary Fig. 16. a, CV curve for PbO_2/Pb^{2+} redox behaviors on FTO electrode at 10 mV s^{-1} in a three-electrode configuration using an electrolyte containing 3 M CH_3SO_3H and 0.5 M PbO, and **b,** the corresponding optical transmittance at 460 nm through a spectroelectrochemical cell.

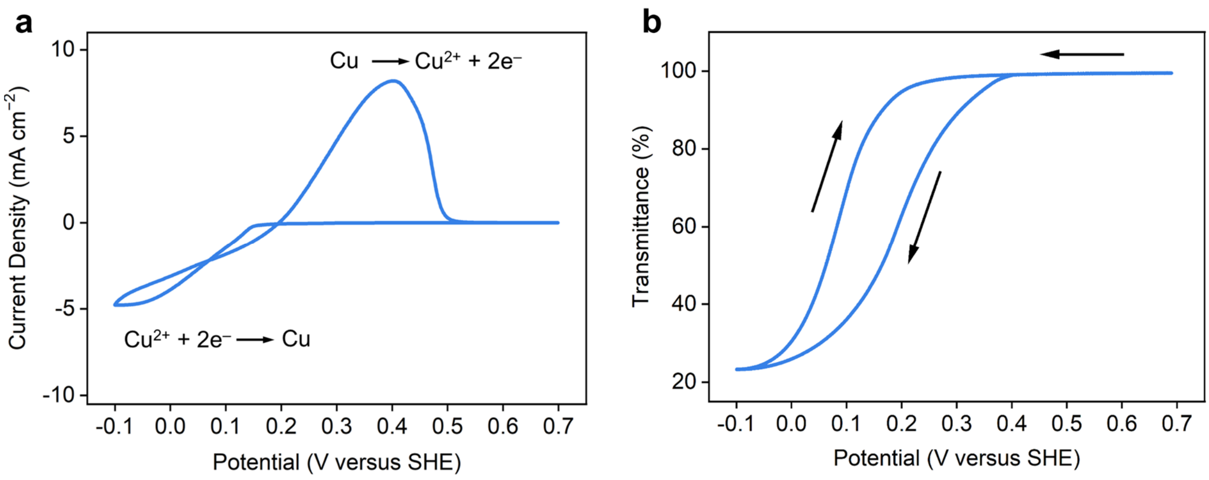

Supplementary Fig. 17. a, CV curve for Cu^{2+}/Cu redox behaviors on FTO electrode at 5 mV s^{-1} in a three-electrode configuration using an electrolyte containing 0.5 M H_2SO_4, 0.5 M $MnSO_4$ and 0.1 M $CuSO_4$ and **b,** the corresponding transmittance at 460 nm through a spectroelectrochemical cell.

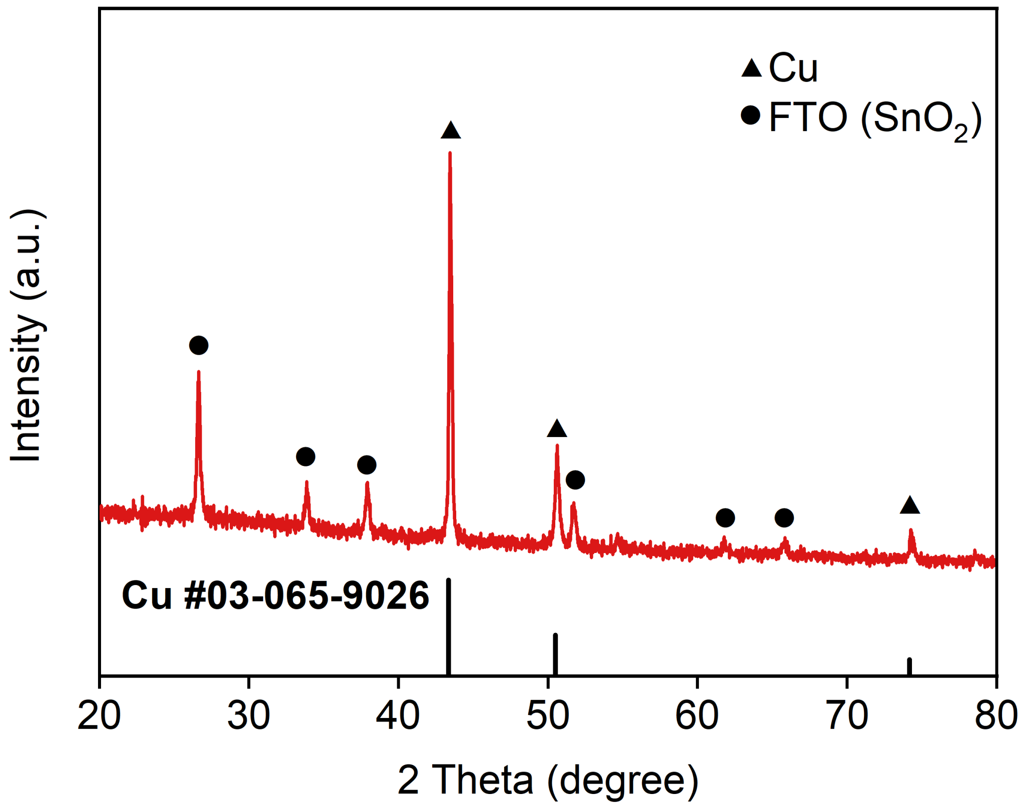

Supplementary Fig. 18. XRD pattern of the deposited Cu on FTO electrode.

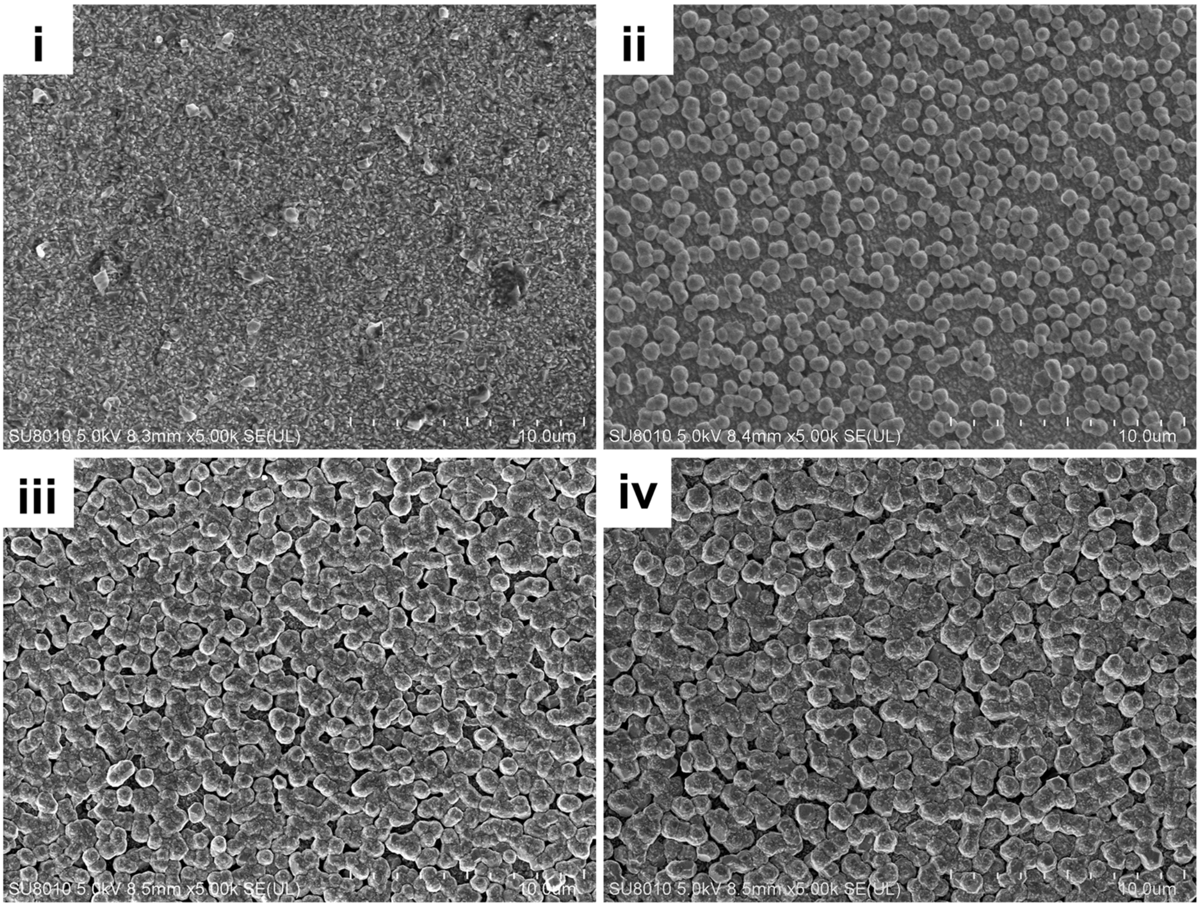

Supplementary Fig. 19. SEM images of FTO electrode before depositing Cu (i) and after deposition at −0.1 V vs. SHE in a three-electrode configuration for 30 s (ii), 60 s (iii), and 180 s (iv).

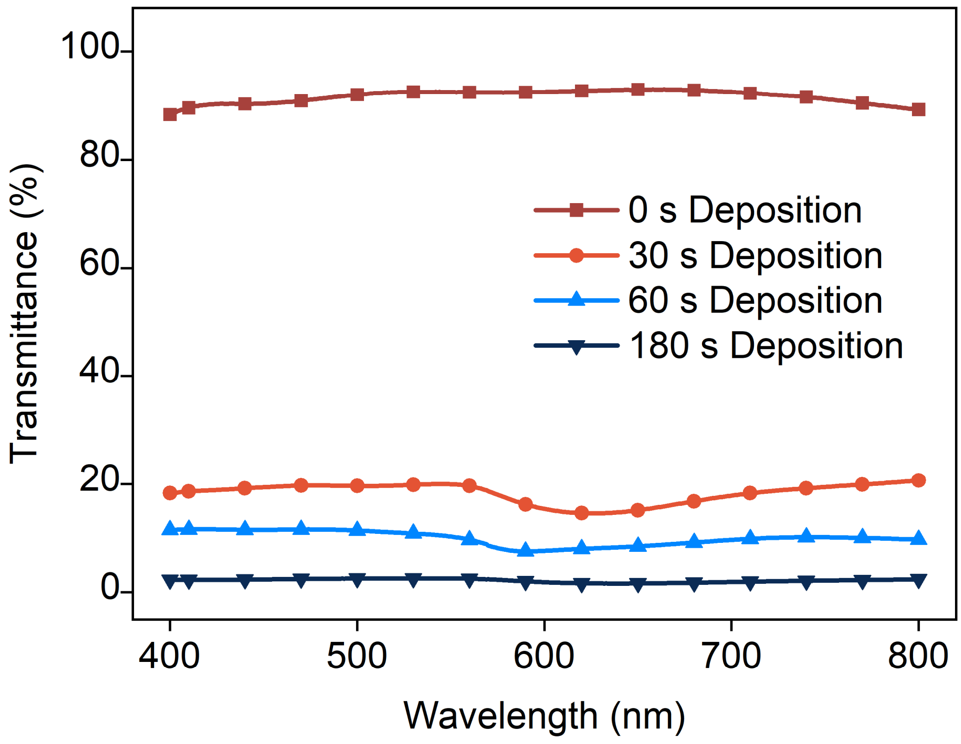

Supplementary Fig. 20. UV-Vis transmittance spectra of FTO electrode before depositing Cu (i) and after deposition at −0.1 V vs. SHE in three-electrode configuration for 30 s (ii), 60 s (iii), and 180 s (iv) using electrolytes containing 0.5 M H_2SO_4, 0.5 M $MnSO_4$, and 0.1 M $CuSO_4$ respectively, in the wavelength range between 400 nm and 800 nm.

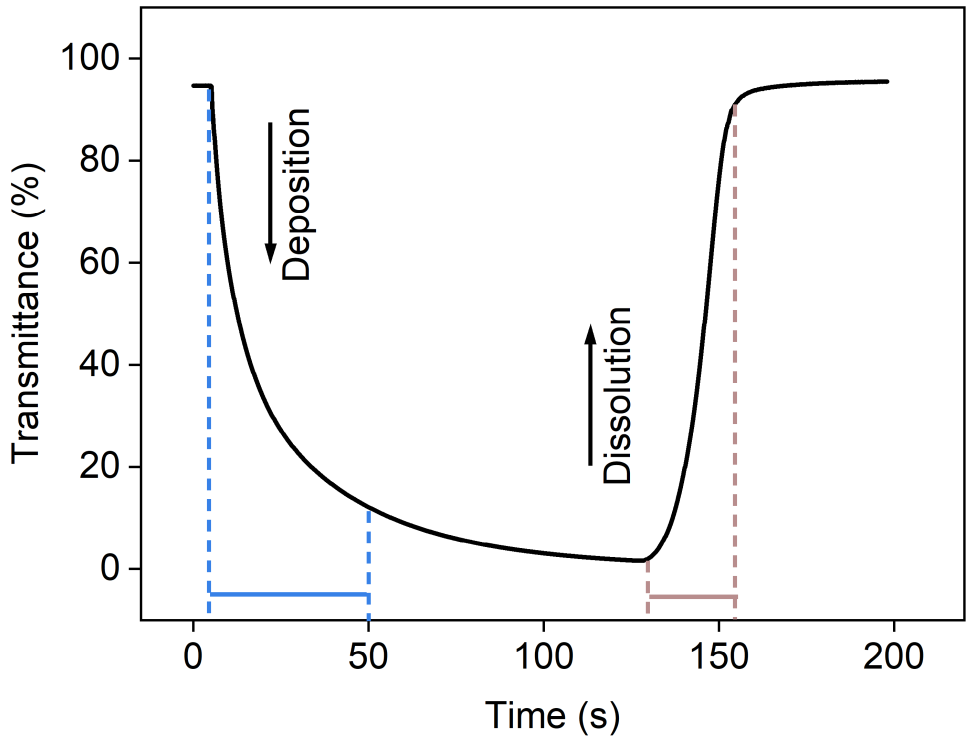

Supplementary Fig. 21. Transmittance spectra at 460 nm of one reversible cycle between Cu deposition and dissolution under alternating redox potential between −0.1 V and 0.7 V vs. SHE in a three-electrode configuration.

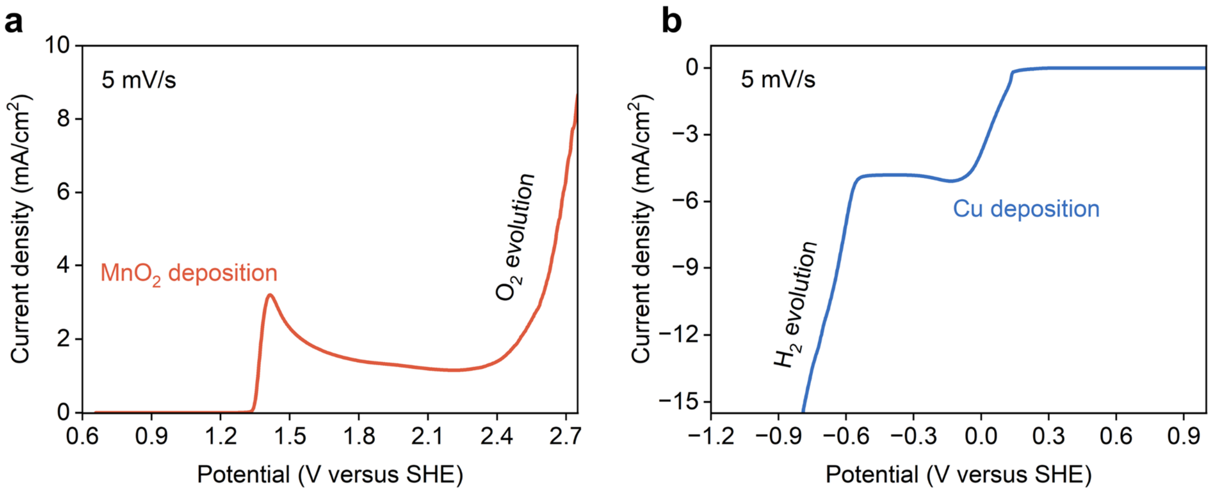

Supplementary Fig. 22. a, Anodic and **b,** Cathodic scan on FTO electrode in a 0.5 M H_2SO_4 + 0.5 M $MnSO_4$ + 0.1 M $CuSO_4$ electrolyte at a scan rate of 5 mV s^{-1}.

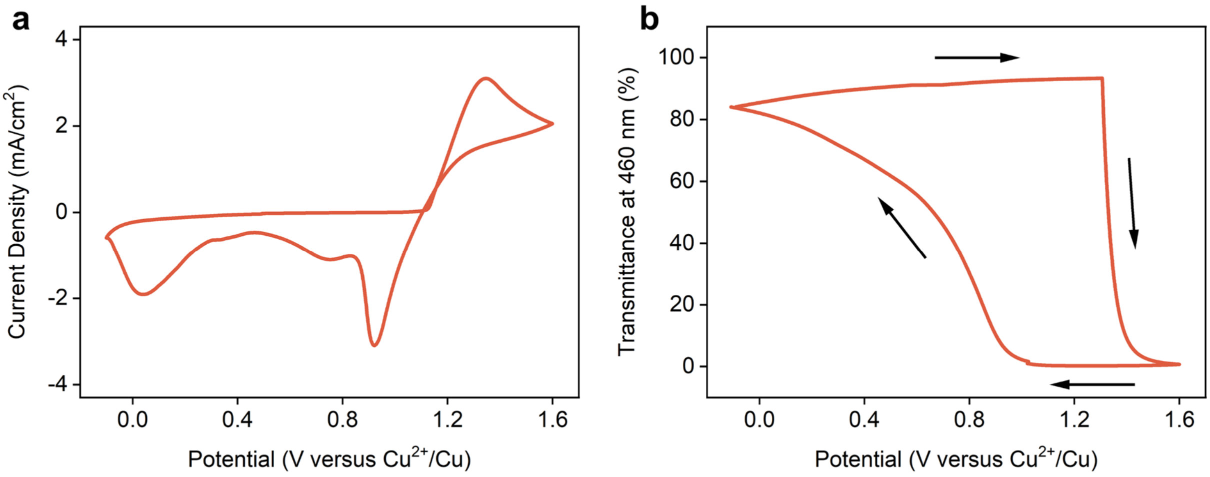

Supplementary Fig. 23. a, CV curve for EECD at 5 mV s^{-1} and **b,** the corresponding *in situ* transmission at 460 nm.

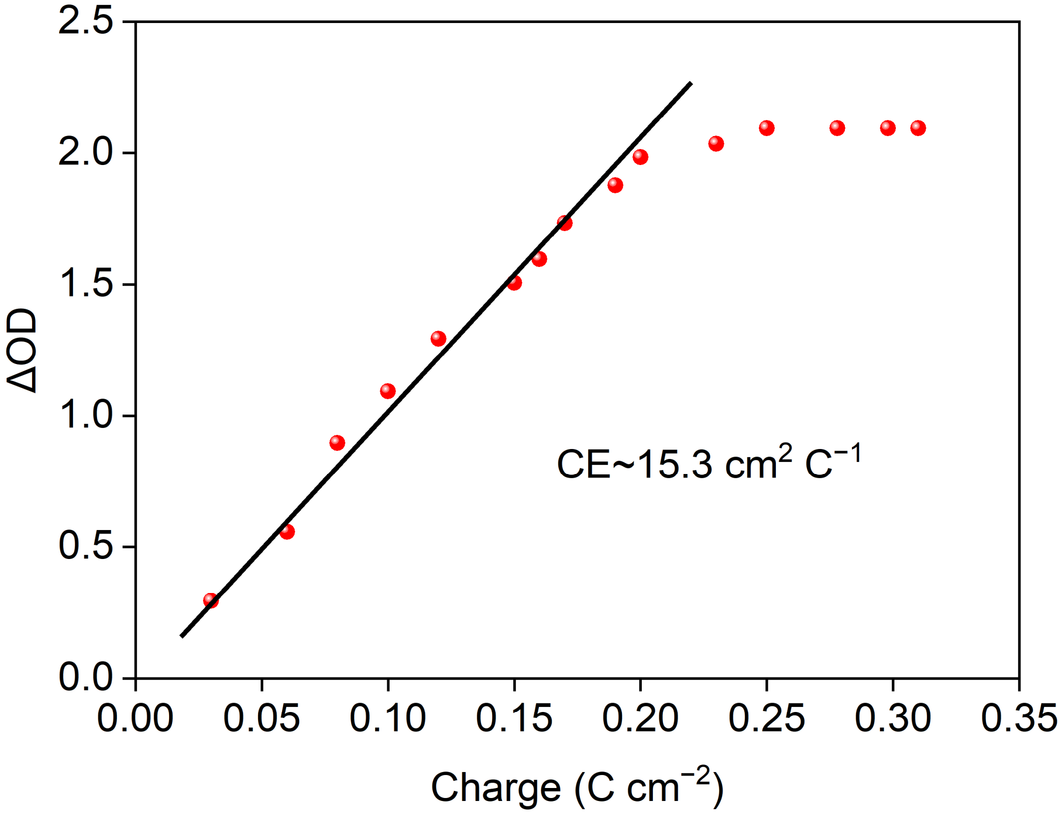

Supplementary Fig. 24. Coloration efficiency of the as-assembled EECD.

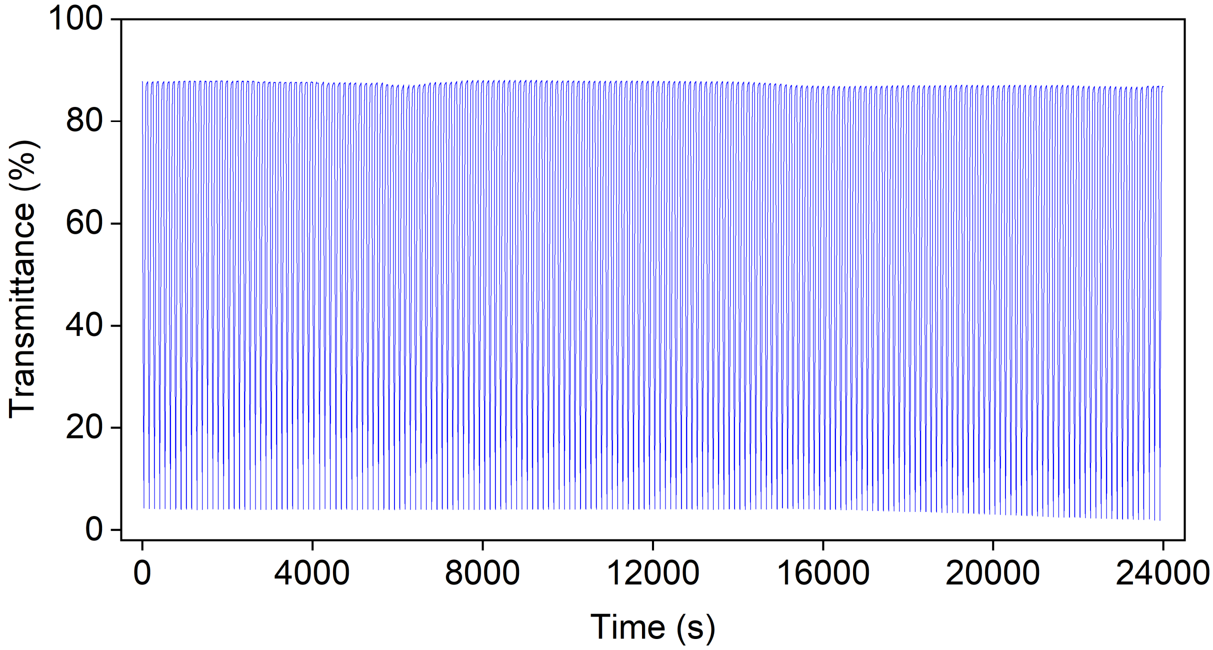

Supplementary Fig. 25. The long-term stability of the EC device, which utilizes a MnO_2/Mn^{2+} redox system for the EC electrode and incorporates a copper frame serving as the counter electrode, has been successfully achieved. This process involves coloring the device at a potential of 1.55 V for a duration of 30 seconds, followed by bleaching at 0 V for a period of 90 seconds.

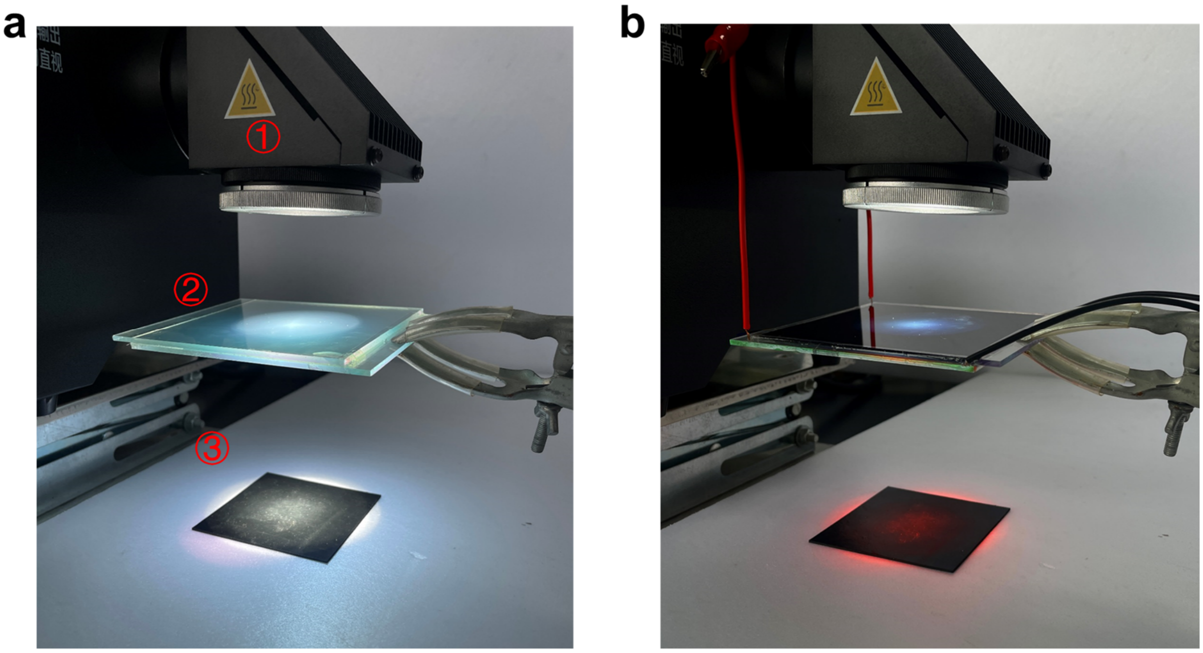

Supplementary Fig. 26. Photographs illustrate the measurement apparatus for thermal images, with component 1 as the xenon lamp, 2 as the 10 cm × 10 cm EECD, and 3 as the endothermic sample.

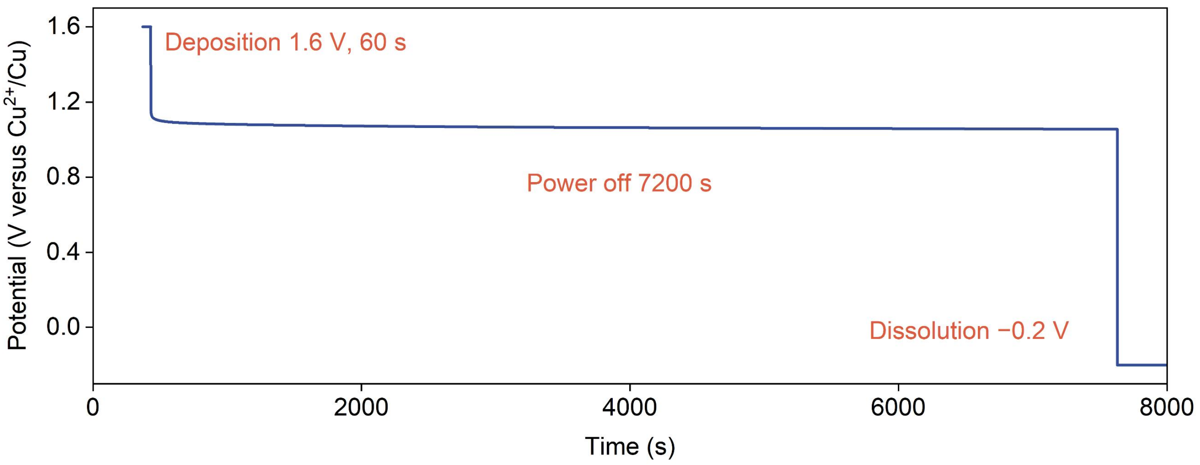

Supplementary Fig. 27. Plot of voltage vs. time with applied deposition at 1.6 V, power off, and dissolution at −0.2 V.

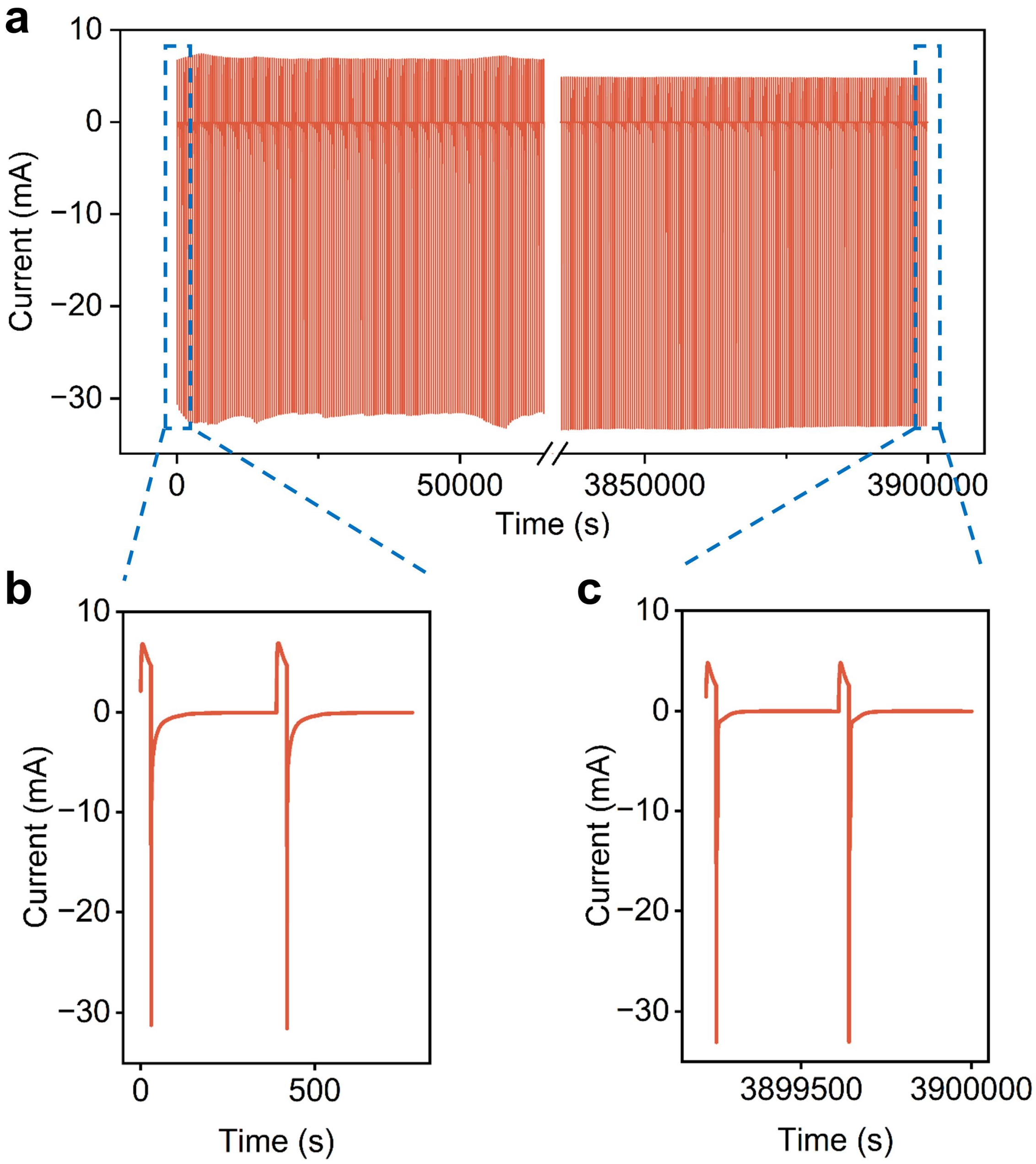

Supplementary Fig. 28. CV curves of EECD sample cycling for 10000 times with voltage switching between 1.6 V for 30 s and −0.2 V for 6 min.

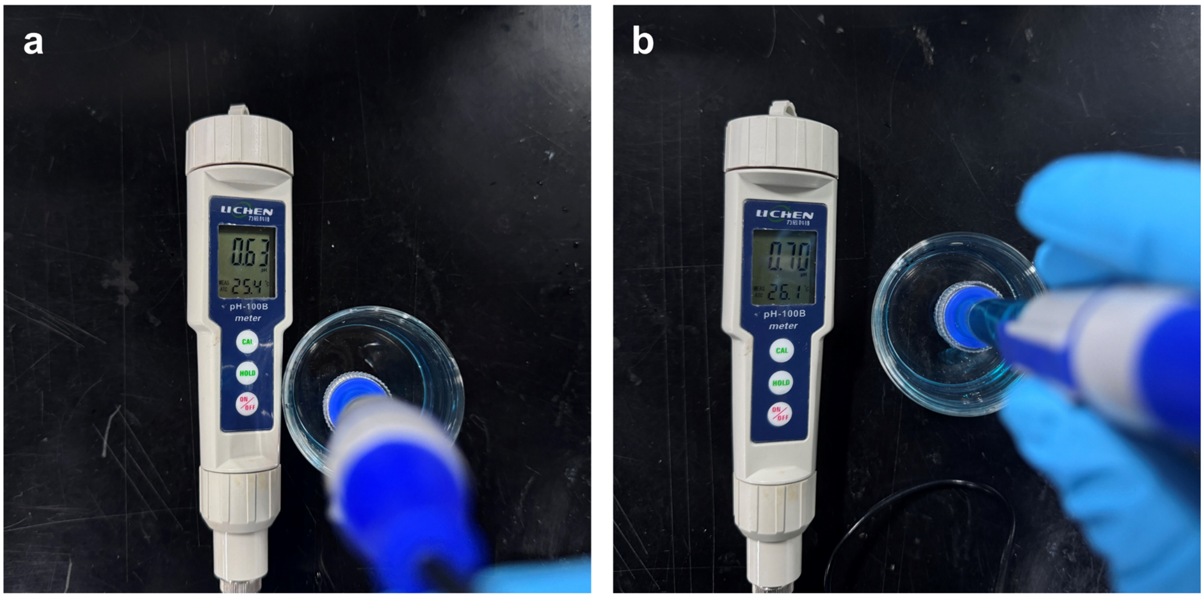

Supplementary Fig. 29. pH value of the electrolyte **a,** before cycling and **b,** after 5000 cycles.

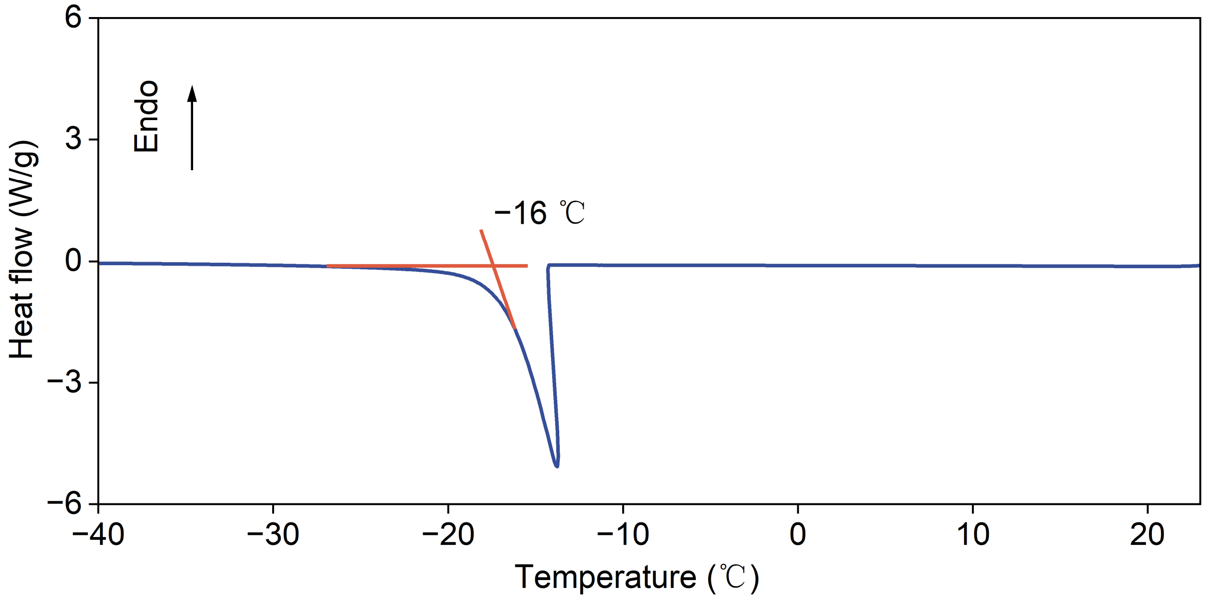

Supplementary Fig. 30. Freezing points of EECD's electrolyte (containing 0.5 M H_2SO_4, 0.5 M $MnSO_4$, and 0.1 M $CuSO_4$) measured by differential scanning calorimetry (DSC) measurement using a liquid nitrogen cooling system.

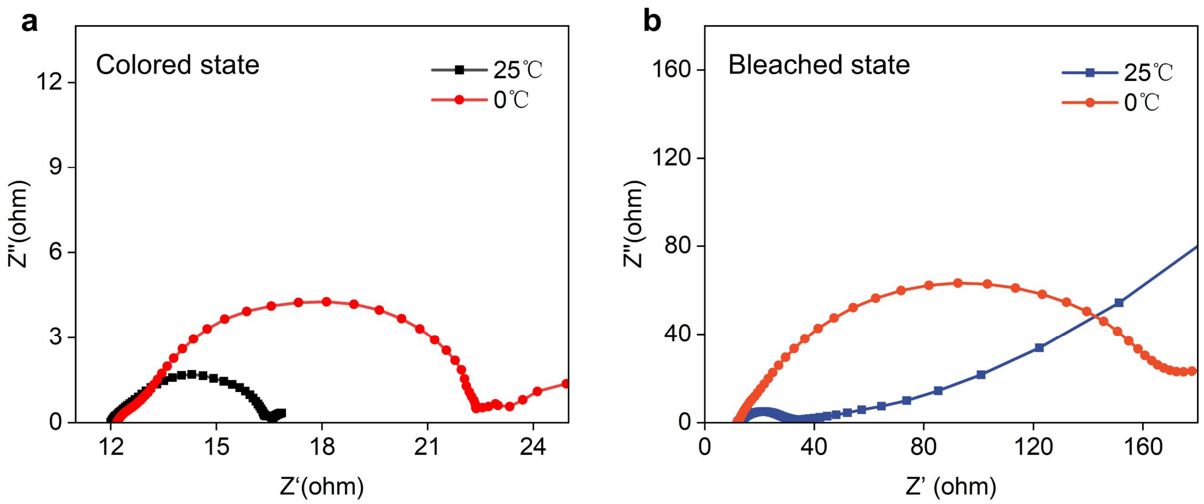

Supplementary Fig. 31 Electrochemical impedance spectroscopy (EIS) profiles of MnO_2 at **a,** the bleached state and **b,** the colored state at different temperatures.

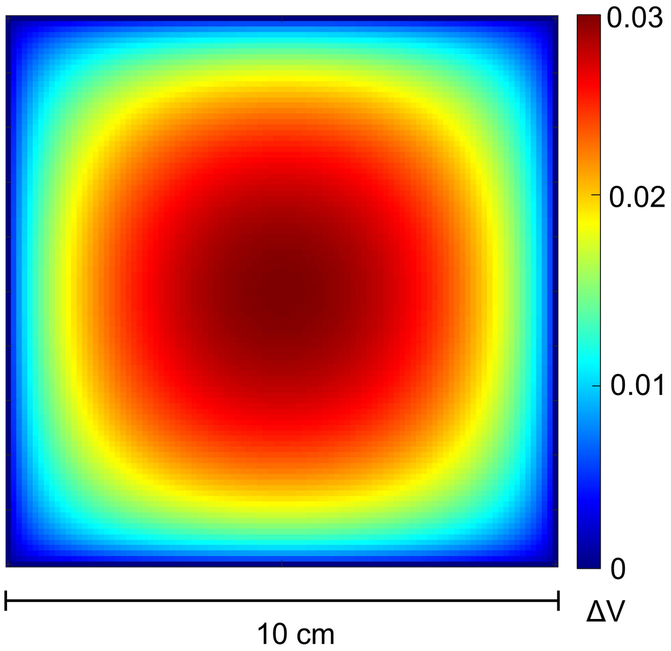

Supplementary Fig.32. Modeled voltage drop across the FTO-coated glass during EECD switching.

The voltage distribution across the FTO-coated glass is provided in Supplementary Fig.33, which was calculated by integrating Ohm's law over the x-y plane of the FTO-coated glass, according to the following Eq (1):

$$\Delta V = \frac{J\rho}{2t}\sqrt{\left[\left(\frac{L}{2}\right)^2 - x^2\right]\left[\left(\frac{L}{2}\right)^2 - y^2\right]} \qquad (1)$$

where J is the current density, ρ is the resistivity of FTO glass (~8×10^{-4} Ω·cm), t is the film thickness of FTO coating (~1 μm), L is the electrode length, and x and y are positions on the FTO surface defined by a Cartesian coordinate system with the origin at the geometric center of the FTO glass. The parameter J represents the maximum current density experimentally needed to switch EECD at 1.6 V, which was determined to be 0.24 mA cm^{-2}. The calculated ΔV is 0.03 V between the edge and the center. For the 40 × 40 cm^2 demo, the ΔV from the edge to the center is about 0.26 V based on calculation.

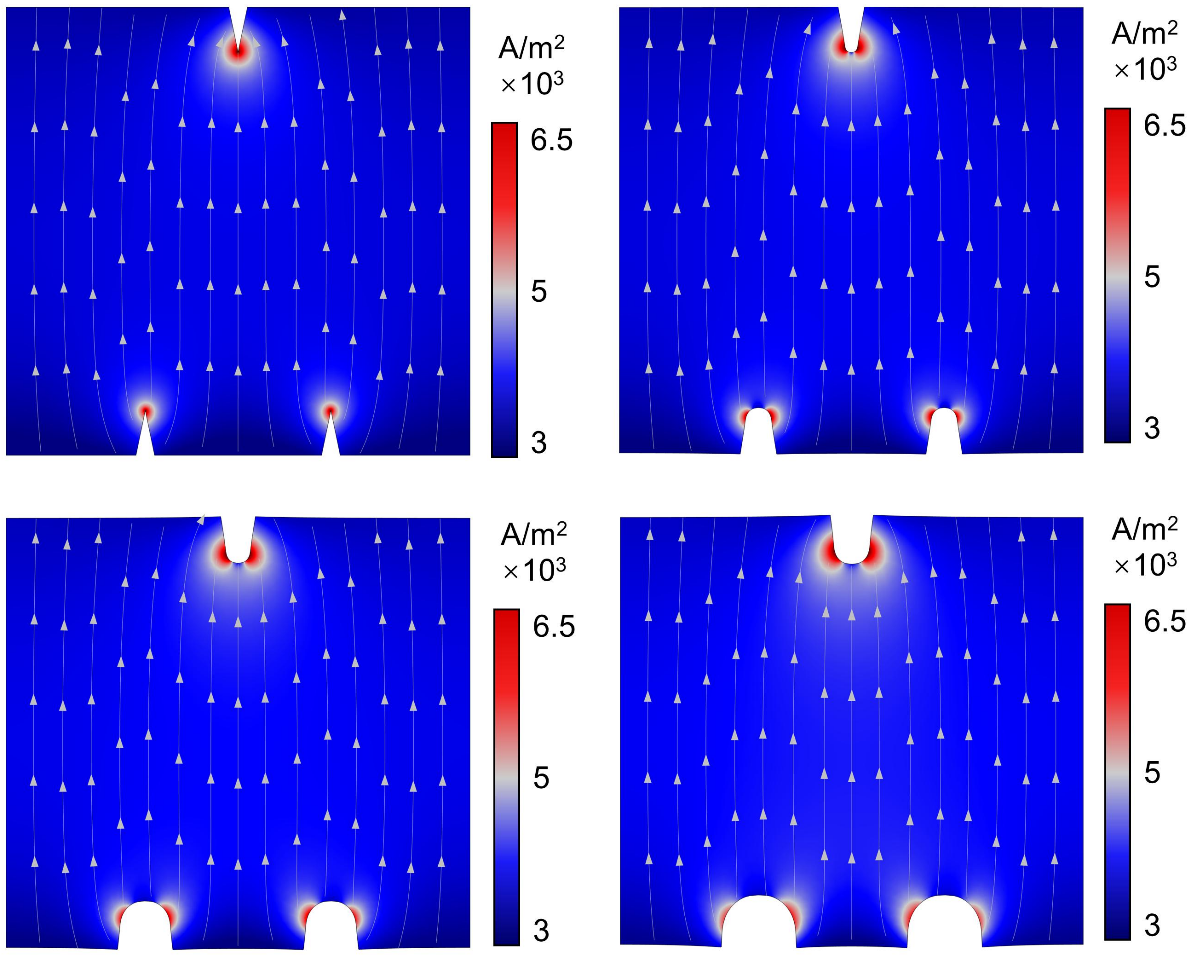

Supplementary Fig. 33. COMSOL simulations of disordered positive and negative electrode interaction during the dual deposition process.

The simulations were carried out in a 2D model. The Cu layer deposits on the top FTO substrate as the anode, and MnO_2 deposits on the bottom FTO substrate as the cathode. Two configurations (one bulge at the top and two at the bottom vs. two bulges at the top and two at the bottom) were simulated. The simulations in Supplementary Fig.27 show the electric field variation of one bulge at the top and two at the bottom. The MnO_2 layer helps homogenize the ion flux and electric field on both electrodes, allowing the uniform plating of both Cu metal and MnO_2

Finite element method (FEM) simulations were carried out by the Cell model of the software COMSOL. Current transmitting simulation was performed by Secondary Current Distribution with the governing Eqs. (2)-(5):

$$\nabla \cdot i_l = Q_l, i_l = -\sigma_l \nabla \phi_l \quad (1)$$

$$\nabla \cdot i_s = Q_l, i_s = -\sigma_s \nabla \quad (2)$$

In the equations, l denotes liquid (electrolyte), s denotes solid (electrode), i is the current intensity density, ϕ is the potential and σ is the electric conductivity.

For electrode kinetics, the local current density is expressed by:

$$i_{loc} = i_0 \left(\frac{(\alpha_a + \alpha_c)F}{RT}\right) \eta \quad (3)$$

In this equation, i_0 is the exchange current density, α_c denotes the cathodic charge transfer

coefficient, α_a is the anodic charge transfer coefficient, T is the temperature, η is the activation overpotential which is determined by φ and the equilibrium potential E_{eq}, and F is the Faraday constant and R is the ideal gas constant.

Deformation of the electrode surface was carried out by deformed geometry and moving meshes and determined by the thickness of deposing species:

$$s = \frac{M}{\rho} c_s, \frac{dc_s}{dt} = \frac{\nu \, i_{loc}}{n F} \quad (4)$$

M is the molar mass, ρ is the density of the species, c_s is the concentration of the species, n is the number of electrons participating in reaction and ν is the stoichiometric coefficients.

The electric field intensity in the electrolyte can be calculated by:

$$\mathrm{E}_l = \nabla \phi_l \quad (5)$$

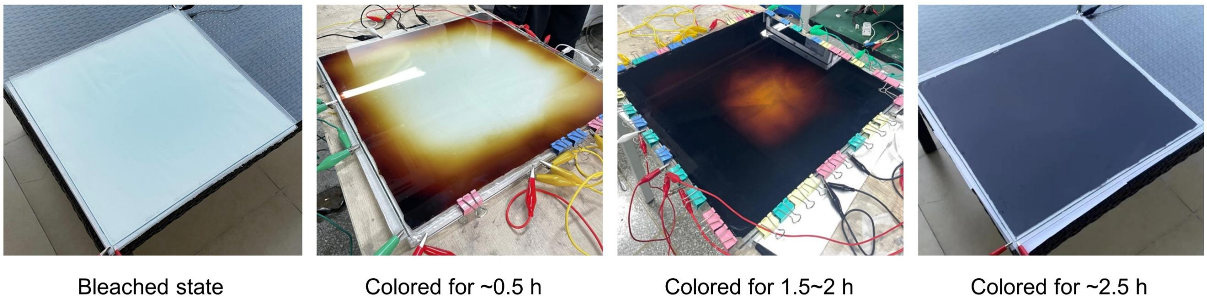

Supplementary Fig. 34. Photographs of the coloration process for 40 × 40 cm^2 EECD.

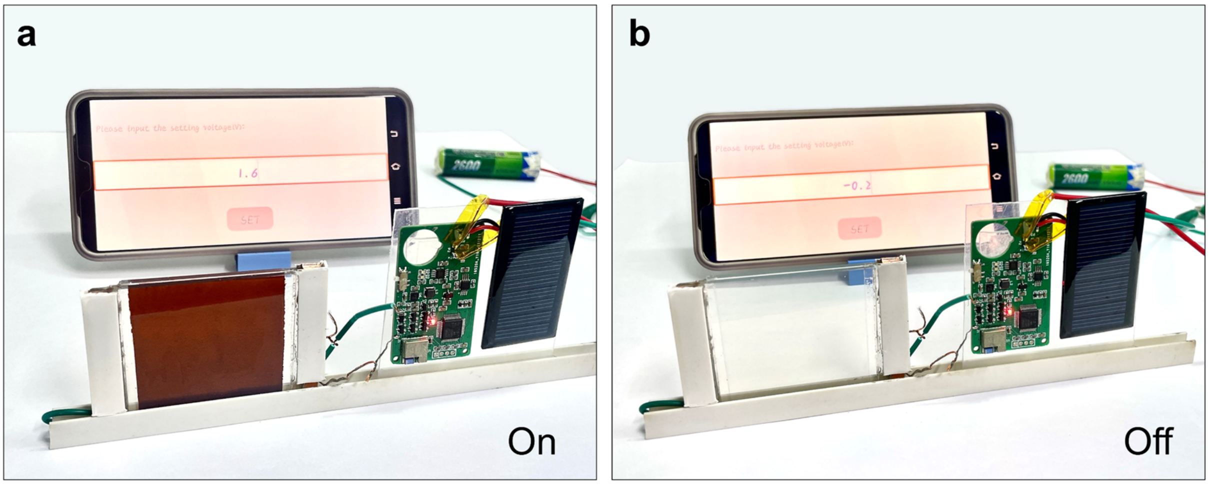

Supplementary Fig. 35. Photographic images of a EECD smart window at colored (a) and bleached (b) states, which was connected to a solar cell and a power management module. The color change was controlled by the App in a mobile phone through Wi-Fi.

Supplementary Table 1. L*, a*, and b* change with different deposition times of depositing MnO_2 on FTO electrode.

**Time (s)**	**L***	**a***	**b***
0 s	93.74	0.04	2.25
30 s	76.90	3.08	36.00
180 s	47.03	26.12	69.06
300 s	23.92	31.99	40.35
600 s	4.81	1.34	-0.02

The data are the average L*, a*, and b* values of different spots (*i.e.,* edge and center) on the MnO_2/FTO electrode.

Cost estimation of EC windows

The cost estimation of EC windows was implemented to EECD and a typical EC device, which all had a 5-layer structure consisting of a TCO electrode, an EC film (usually it's tungsten oxide-WO_3), an ion-conducting electrolyte, an ion storage layer, and a second TCO electrode. Some of the details of the production process involved in calculations refer to previously reported literature[1-4]. Here we also refer to the methods used in the previously reported work on cost estimation[5-7]. Additionally, the following cost estimates of EC windows here are based on an annual production rate of 100,000 units, including capital cost (*e.g.*, facility investment and depreciation, etc.), materials cost, and overhead cost (*e.g.*, labor cost and utilities, etc.). The overhead cost of the production process was estimated based on reasonable assumption and other costs are calculated according to the set output.

Supplementary Table 2. Comparing processes of EECD and typical EC windows.

	**Processes**
Manufacturing lines shared by EECD and typical EC windows	TCO substrate cleaning, preparation of electrolyte formulation, injection of electrolyte, encapsulation
No electrode coating process involved in the manufacturing line of EECD	--
Electrode coating process involved in the manufacturing line of typical EC windows	Sputtering the EC layer and counter-electrode layer
Estimated capital investment of EECD production line	--
Estimated capital investment of typical EC windows	Magnetron sputtering equipment, vacuum system, gas and exhaust treatment system, etc.

As an important factor of production capacity, the production rate highly depends on the technologies used for the manufacturing line. Table 1 compares the common processes and differences between the EECD and typical EC. Sputtering techniques used for the preparation of EC layer and counter electrode materials are considered as the rate-dominating steps in typical EC windows manufacturing lines. In addition, the costly facility takes nearly 60% of capital investment.

Supplementary Table 3. Capital cost of EECD and typical EC windows along with facility depreciation

	**Investment US$**	**Depreciation and maintenance cost US$/m^2**	**Capital cost US$/m^2**
EECD	2500000	15	40
typical EC windows	8000000	40	120

EECD and typical EC windows differ greatly in calculated capital costs. The initial capital cost of EECD was lower because simple fabrication without expensive coating techniques was lower than the magnetron sputtering machines (vacuum and cooling systems are also required) used in typical EC windows. The capital cost also includes the cost of equipment depreciation and later maintenance.

Supplementary Table 4. Estimation of materials cost for EECD and typical EC windows.

	**EECD**	**typical EC window**
expected materials cost US$	3060000	4050000
materials use ratio	80%	80%
materials US$/m^2	38.3	50.6

The cost of materials was estimated based on the amount of materials used in a shipment of 10,000 units a year, including FTO/glass, electrolytes, and target materials. The relatively high materials cost of typical EC windows was due to the target materials consumption for the preparation of the EC layer and expensive organic electrolyte. The materials cost of EECD was lower because of cheap aqueous electrolyte and EC-layer-free.

Supplementary Table 5. The labor cost of a manufacturing line for EC windows.

	Employee number	Average wage US$/year	Cost US$/year	Labor cost US$/m^2
EECD	35	40000	1400000	1.4
typical EC windows	105	40000	4200000	4.2

The manufacturing line of typical EC windows requires sputtering the EC layer and counter-electrode layer. Compared to EECD, which only requires electrolyte preparation and packaging, it requires three times as many employees. Take EECD production as an example, one manufacturing line requires three groups to switch every 8 h for one day. There are 10 operators in one group; 3 technicians and 2 managers to direct the operators, the number of employees is around 35 in total. The average wage is assumed by considering the balance between developing country such as China and developed country such as USA[6].

Supplementary Table 6. Estimated overhead cost for two types of EC windows.

Cost component	Typical EC Windows cost (US$/m^2)	EECD windows cost (US$/m^2)
Facilities (*e.g.* rent)	0.2	0.01
Utilities (electricity, water)	0.3	0.01
Labor	4.2	1.4
Maintenance	0.1	0.03
Sum	4.8	1.45

The overhead cost is estimated by the sum of facilities, utilities, labor, and maintenance fees.

Supplementary Table 7. Estimation of cost for EC windows

	Capital Cost US$/m^2	Cost of Materials US$/m^2	Overhead Cost US$/m^2	Sum US$/m^2
EECD	40	38.3	1.45	79.8
typical EC windows	120	50.6	4.8	175.4

Supplementary Table 8. Comparison of current state-of-the-art EC films and devices. (The data presented are the performance in the visible light region).

Ref.	Device or electrode	Achievable color states	ΔT(visible)	Switching speed	bistability	Cycle life
8	device	Transparent, brown	73.5%	21.6 s/26.6 s	--	--
	film electrode		90.8%	4.4 s/12.6 s	5000 s	300
9	device	Black, transparent, gray shades	>70%	180 s tint to 0.01%/--	35 h (after tint for 360 s)	5000
10	device	Transparent, blue	65%	17 s/15 s	--	--
	film electrode		70%	12 s/5 s	--	6000
11	device	Black, transparent, gray shades	75%	180 s tint to 5%/70 s	24 h (applied small current)	5500
12	device	Bright, cool, dark	74.5%	60 s/60 s	--	500
	film electrode		95.5%	35.1 s/9.6 s	3600 s	2000
13	film electrode	Bright, cool, dark	89.1%	52.6 s/9.5 s	3600 s	2000
14	device	Black, transparent	65%	60 s/~90 s	--	4000
15	device	Blue, transparent	62%	17 s/--	--	100
**This work**	**device**	**Black, brown, yellow, transparent**	**85%**	**17 s/147 s**	**7200 s**	**10000**

ΔT of the film-based electrodes was measured after the blank sample (*i.e.* TCO electrode) was deducted. Parts of the data shown in the table were measured from the graph in the references and marked by the symbol "~"

References

1. Papaefthimiou, S., Syrrakou, E., Yianoulis, P. Energy performance assessment of an electrochromic window. *Thin Solid Films* **502**, 257-264 (2006).
2. Syrrakou, E., Papaefthimiou, S., Yianoulis, P. Environmental assessment of electrochromic glazing production. *Sol. Energy Mater. Sol. Cells* **85**, 205-240 (2005).
3. Khaled, K., Berardi, U. Current and future coating technologies for architectural glazing applications. *Energy Build.* **244**, 111022 (2021).
4. Papaefthimiou, S. Chromogenic technologies: Towards the realization of smart electrochromic glazing for energy-saving applications in buildings. *Adv. Build. Energy Res.* **4**, 77-126 (2010).
5. Schmidt, O., Hawkes, A., Gambhir, A., Staffell, I. The future cost of electrical energy storage based on experience rates. *Nat. Energy* **2**, 17110 (2017).
6. Cai, M. L. et al. Cost-performance analysis of perovskite solar modules. *Adv. Sci.* **4**, 1600269 (2017).
7. Chang, N. L. et al. A manufacturing cost estimation method with uncertainty analysis and its application to perovskite on glass photovoltaic modules. *Prog. Photovoltaics* **25**, 390-405 (2017).
8. Lei, P. Y. et al. An electrochromic nickel phosphate film for large-area smart window with ultra-large optical modulation. *Nano Micro Lett.* **15**, 34 (2023).
9. Strand, M. T. et al. Polymer inhibitors enable >900 cm^2 dynamic windows based on reversible metal electrodeposition with high solar modulation. *Nat. Energy* **6**, 546-554 (2021).
10. Cheng, W. et al. Photodeposited amorphous oxide films for electrochromic windows. *Chem-US* **4**, 821-832 (2018).
11. Barile, C. J. et al. Dynamic windows with neutral color, high contrast, and excellent durability using reversible metal electrodeposition. *Joule* **1**, 133-145 (2017).
12. Zhang, S. L., Cao, S., Zhang, T. R., Lee, J. Y. Plasmonic oxygen-deficient TiO_{2-x} nanocrystals for dual-band electrochromic smart windows with efficient energy recycling. *Adv. Mater.* **32**, 2004686 (2020).
13. Cao, S. et al. A visible light-near-infrared dual-band smart window with internal energy storage. *Joule* **3**, 1152-1162 (2019).
14. Islam, S. M., Hernandez, T. S., McGehee, M. D., Barile, C. Hybrid dynamic windows using reversible metal electrodeposition and ion insertion. *Nat. Energy* **4**, 223-229 (2019).
15. Li, H. Z., McRae, L., Firby, C. J., Elezzabi, A. Y. Rechargeable aqueous electrochromic batteries utilizing Ti-substituted tungsten molybdenum oxide based Zn^{2+} ion intercalation cathodes. *Adv. Mater.* **31**, 1807065 (2019).
